# CH−π interactions confer orientational flexibility in protein–carbohydrate binding sites

**DOI:** 10.1016/j.jbc.2025.110379

**Published:** 2025-06-14

**Authors:** Allison M. Keys, David W. Kastner, Laura L. Kiessling, Heather J. Kulik

**Affiliations:** 1 Computational and Systems Biology Program, Massachusetts Institute of Technology, Cambridge, Massachusetts, USA; 2 Department of Chemical Engineering, MIT, Cambridge, Massachusetts, USA; 3 Department of Chemistry, MIT, Cambridge, Massachusetts, USA; 4 Department of Biological Engineering, MIT, Cambridge, Massachusetts, USA; 5 The Broad Institute of MIT and Harvard, Cambridge, Massachusetts, USA; 6 Koch Institute for Integrative Cancer Research, MIT, Cambridge, Massachusetts, USA

**Keywords:** protein-carbohydrate binding, CH−π interactions, carbohydrate-aromatic interaction, D-galactose, metadynamics, molecular dynamics simulations, interaction dynamics, binding specificity, processivity

## Abstract

Protein–carbohydrate binding plays an essential role in biological processes including cellular recognition and immune signaling. However, glycans are hydrophilic with limited hydrophobic surfaces, a challenge for selective recognition by proteins. CH–π stacking interactions are pervasive in protein–carbohydrate binding sites and have emerged as critical drivers of protein–carbohydrate recognition. These interactions are highly favorable and have a broad orientational landscape. However, it is unknown how the orientations of CH−π stacking interactions are influenced by the protein environment; their functional interplay with hydrogen bonds in protein–carbohydrate binding is also unclear. Here, we employ well-tempered metadynamics simulations to obtain binding free energy landscapes for a set of protein−β-D-galactoside complexes with CH–π stacking interactions. Our data show that the favored orientation of a CH−π stacking interaction is controlled by the location of hydrogen bonds in the protein binding site. Complexes with extended carbohydrate ligands that form additional hydrogen bonds have more specific orientational dependencies, while protein variant complexes with fewer hydrogen bonds have broader free energy landscapes with glycan ligands adopting multiple CH−π stacking interaction orientations. We also show that forming multiple CH−π stacking interactions facilitates the dynamics necessary for the translocation of oligosaccharide ligands within a processive enzyme. Our findings underscore the cooperative nature of hydrogen bonds and CH−π stacking interactions, demonstrating that tuning the number and positions of these interactions through evolution or protein engineering can alter ligand recognition or support ligand movement.

Selective protein–carbohydrate recognition is essential for many biological processes, including immunological responses and the biosynthesis and degradation of glycans (1, 2, 3, 4, 5). However, the monosaccharides that comprise oligosaccharides have high structural similarity and hydrophilicity, making them difficult ligands for selective recognition by proteins. As a result, glycan-protein complexes have relatively weak protein binding affinities, especially when the ligand is a monosaccharide, disaccharide, or trisaccharide (*K*_d_ = 10^−3^–10^−6^ M) (6, 7, 8). Despite this weak affinity, many proteins achieve high selectivity for their carbohydrate ligands (9, 10). However, the mechanism that drives this selectivity is not well understood. Thus, elucidating the role of the noncovalent interactions that drive protein–carbohydrate binding affinity and selectivity may enable advanced exploration and engineering of novel protein–carbohydrate binding sites.

Carbohydrate-binding proteins are often analyzed using glycan arrays (11, 12, 13, 14, 15, 16), which can be used to develop binding profiles and identify the protein’s glycan determinant(s), *i.e.*, a carbohydrate sequence that is selectively recognized by the protein (7). Other experimental techniques, including bio-layer interferometry (17, 18, 19, 20), nuclear magnetic resonance (NMR) (21, 22, 23), and isothermal titration calorimetry (24, 25), can then be used to measure binding kinetics and affinities of individual protein–carbohydrate interactions. These experiments coupled with mutational analyses can reveal the contributions of specific amino acids to the binding affinity (24, 26, 27). Although these tools can provide essential insights into protein–carbohydrate interactions, they are limited in their ability to elucidate the conformational dynamics of binding and evaluate the cooperativity of individual noncovalent interactions. An understanding of these issues is required for a deeper understanding of the forces that drive selective recognition by proteins.

Computational tools such as molecular dynamics (MD) simulations can capture protein dynamics and therefore bridge this gap. Direct MD can be coupled with enhanced sampling techniques to efficiently sample the conformational dynamics and geometries of a protein–carbohydrate complex and quantify the free energy of a binding interaction. Metadynamics (MetaD) simulations can reveal the underlying free energy landscape of a binding interaction relative to collective variables (CVs) that define binding (*e.g.*, ligand orientation) (28, 29, 30, 31). When coupled with energy decomposition schemes, these methods provide a comprehensive understanding of the roles that individual amino acids play in binding affinity and selectivity (32, 33).

Protein–carbohydrate binding is generally governed by multiple types of noncovalent interactions: hydrogen bonds, metal cation-mediated interactions, and CH−π interactions (6, 34). Networks of hydrogen bonds are prevalent in most carbohydrate binding sites, with multiple amino acids engaging hydroxyl groups on the carbohydrate (35, 36, 37). Some glycan-binding proteins also have cation-mediated interactions, which involve the coordination of carbohydrate hydroxyl groups to a protein-chelated metal ion, typically calcium (34). These interaction types are expected to be primarily electrostatic and are defined by a rigid relationship between their orientation and interaction energy (38). Alternatively, CH−π stacking interactions, which are formed by multiple C–H groups on the carbohydrate that stack on top of aromatic ring(s) in the protein, have both electrostatic and van der Waals energy components (39). These CH−π stacking interactions provide a substantive driving force for binding because they cannot be formed with water in the environment, unlike hydrogen bonding and metal-mediated interactions (6).

Prior studies have demonstrated that not only are aromatic amino acids enriched in protein–carbohydrate binding sites (40), but importantly, the CH−π stacking interactions they form are energetically favorable (39, 41, 42, 43), essential for binding in some protein–carbohydrate complexes (26), and are implicated in reactivity and processivity in enzymes (44, 45, 46, 47). Because CH−π stacking interactions are composed of favorable contacts from multiple C–H groups, they occupy many distinct orientations, with variations in which C–H groups interact and the relative rotation of the aromatic ring(s). We have shown that CH−π stacking interactions with galactose have a broad orientational landscape that can be defined by only two CVs derived from the distance of galactose carbon atoms to the aromatic ring of the amino acid (39). A range of these CVs corresponds to highly favorable interactions (39). However, most knowledge of CH−π stacking interaction energetics and orientational preferences has been derived from small-molecule models (39, 40, 41, 42, 43, 48, 49, 50, 51, 52, 53). As such, there is limited knowledge on the influence of the protein environment on CH−π stacking interaction orientation, even though the broad range of CVs observed in these interactions suggests that the binding environment may play a key role in defining the preferred stacking orientation in that system. An assessment of the influence of the protein environment on the orientational dynamics of CH−π stacking interactions is needed to better understand protein–carbohydrate interaction selectivity.

In this study, we evaluated several protein–carbohydrate complexes, exploring the effect of the quantity and position of hydrogen bonds and CH−π stacking interactions on binding dynamics. We performed well-tempered MetaD (WT-MetaD) simulations on these systems to obtain free energy landscapes of the bound complexes relative to the CH−π stacking interaction orientations, and we carried out MD simulations with energy decompositions to evaluate the enthalpic contributions from all residues. We found that these protein–carbohydrate interactions occupy a range of CH−π stacking interaction orientations and that the breadth of orientations sampled is driven by the number, type, and flexibility of hydrogen bonds present in the binding site. We show that oligosaccharides that form additional hydrogen bonds in comparison to monosaccharides result in greater orientational specificity of binding. Conversely, protein variants that lack key hydrogen bonds result in greater orientational flexibility. Finally, we demonstrate that the formation of multiple CH−π stacking interactions supports a broader range of binding orientations and should enable favorable translocation of ligands in processive enzymes. Our work supports future analysis and engineering of protein–carbohydrate binding sites for selectivity and enzyme function.

## Results

### Galactose CH−π interactions in protein–carbohydrate binding sites

**Figure 1:**
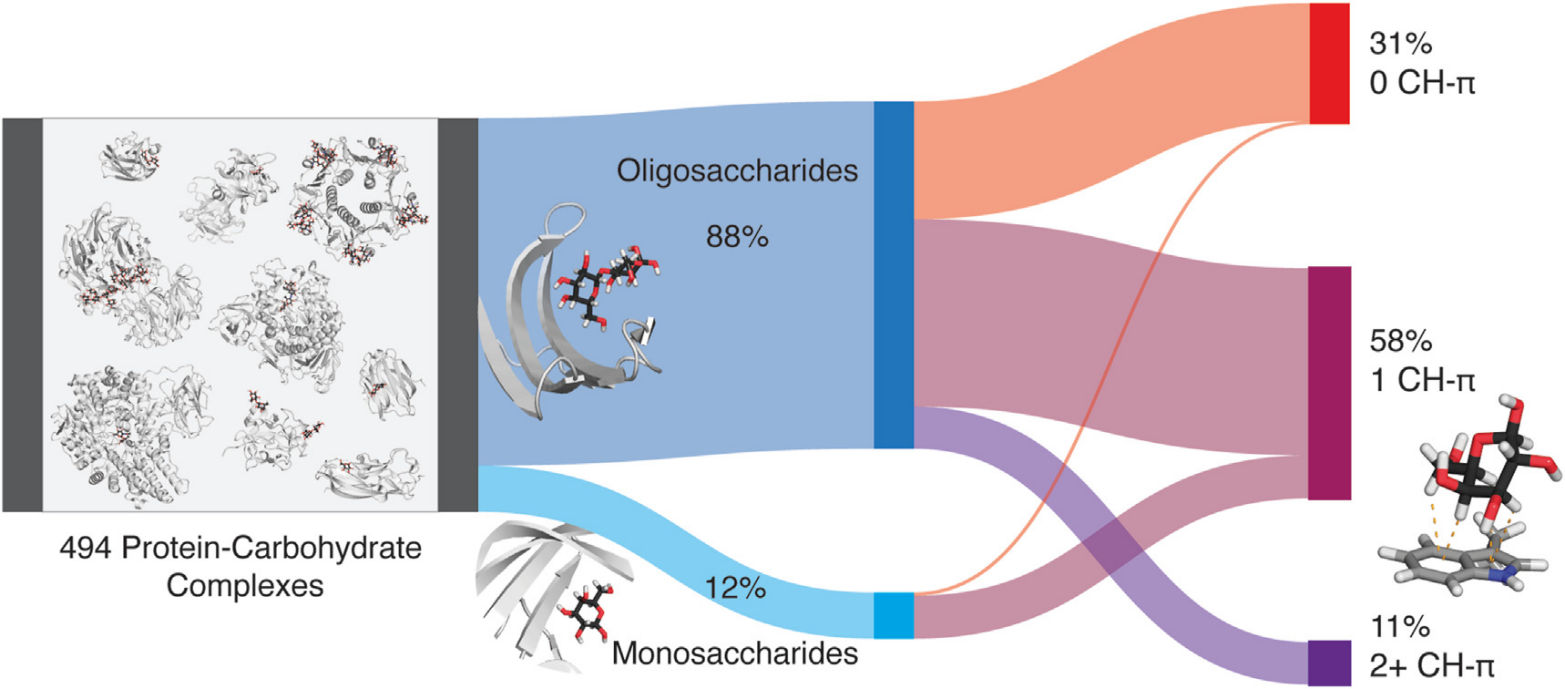
**Sankey diagram depicting dataset generation and analysis.** From *left*, noncovalent protein–carbohydrate interactions to β-D-galactosides in the Protein Data Bank (PDB) are categorized by carbohydrate ligand sequence (monosaccharide or oligosaccharide) and the number of CH–π stacking interactions formed between the carbohydrate ligand and aromatic amino acids in the protein. Atoms are colored as follows: protein carbon atoms in *gray*, carbohydrate carbon atoms in *black*, oxygen in *red*, nitrogen in *blue*, and hydrogen in *white*.

We curated a dataset of noncovalent protein–β-D-galactoside binding interactions using the Protein Data Bank (PDB) (54). In a search on 11/19/2021 that used cutoffs of 2 Å resolution and 0.2 R-factor, we identified 494 well-resolved crystal structures of protein–β-D-galactoside complexes (Fig. 1). We used a 95% sequence identity cutoff to identify duplicate proteins and determined that the full set contained 240 unique mammalian, bacterial, and viral proteins (See Supporting Information Data on Zenodo (55)). This set includes noncovalent complexes with lectins, enzymes, and other carbohydrate-binding proteins bound to a range of β-D-galactoside ligands. Out of the 494 total structures, 436 were bound to oligosaccharides, comprising 88% of the dataset. The other 58 structures contained monomeric β-D-galactose as the ligand (Fig. 1).

We assessed the frequency of CH–π interactions in these binding sites using two features proposed previously: *d*_Cn-Ctr,_ the distance from a galactose carbon atom (Cn) to the centroid (Ctr) of the nearest aromatic ring; and *θ*_Proj-Cn-Ctr_, the angle between the distance vector, *d*_Cn-Ctr_, and the projection of Cn onto the aromatic ring plane (24, 56). Carbohydrate C–H groups are defined as CH–π interactions when *d*_Cn-Ctr_ < 4.6 Å and *θ*_Proj-Cn-Ctr_ < 50°. We then identified carbohydrate residues that engaged in CH–π stacking interactions, which involve the formation of three or more individual CH–π interactions between the carbohydrate and the aromatic ring(s). Of the 494 bound structures, 69% formed a CH−π stacking interaction between an aromatic amino acid and at least one carbohydrate monomer in the ligand (Fig. 1). Comparing oligosaccharides to monosaccharides, we observed a significantly higher prevalence of CH–π stacking interactions in the monosaccharide binding sites (93 *versus* 66%). The higher propensity of CH–π stacking interactions in monosaccharide binding sites suggests that while larger oligosaccharide ligands can experience sufficient binding stabilization solely through hydrogen bonding and metal-mediated interactions, CH–π stacking interactions are more essential in monosaccharide binding.

A small subset of the data, comprising only 53 structures and 39 unique proteins, formed CH–π stacking interactions to more than one carbohydrate residue in an oligosaccharide (Supporting Information Table S1). Of these proteins, 66% were annotated as enzymes, while the other 34% were carbohydrate-binding proteins. Compared to the full dataset, for which only 29% of the proteins were enzymes, this is a significant enrichment (χ^2^ test *p*-value < 0.00001). The propensity of multiple CH–π stacking interactions in enzymes suggests a possible functional basis for the formation of multiple CH–π stacking interactions, which we will revisit in Orientational flexibility of CH–π interactions enables processivity.

### Protein–monosaccharide interaction energetics

**Figure 2:**
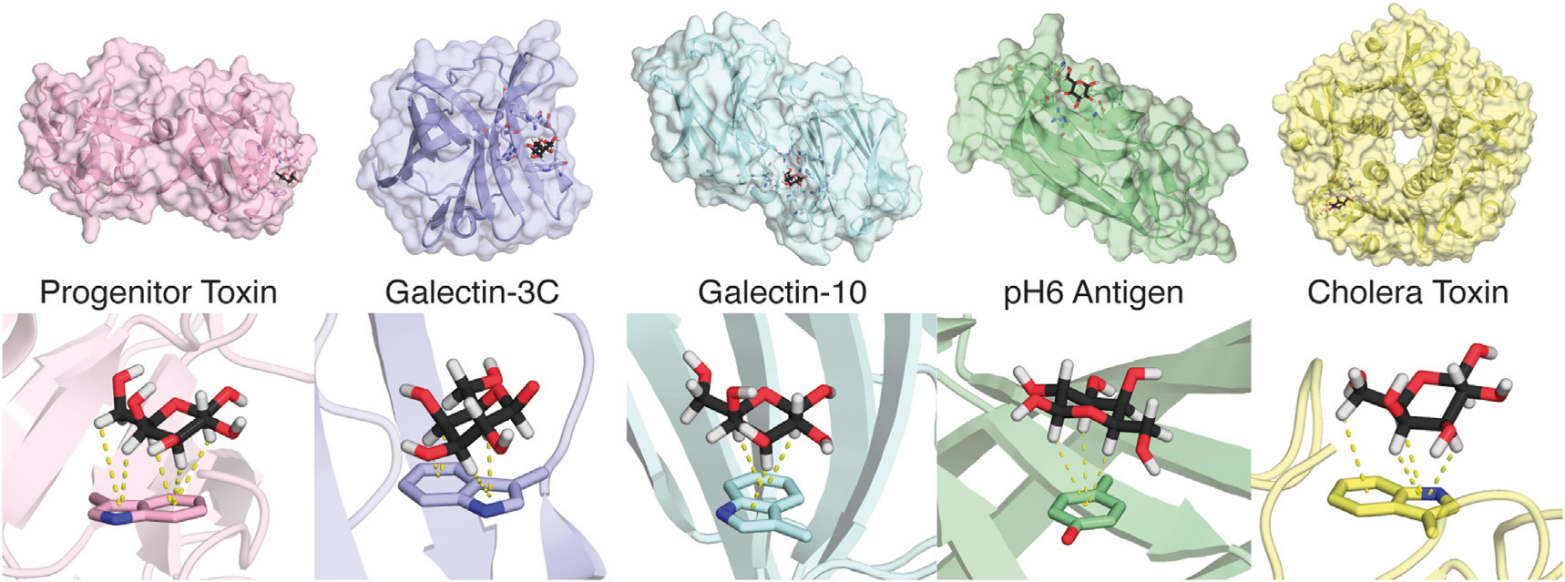
**Visualizations of protein–carbohydrate binding sites and the corresponding CH–π stacking interactions.** (*Top*) Visualization of the five labeled proteins bound to β-D-galactose from *left* to *right*, PDB IDs: Progenitor toxin, 3AH4 (57); galectin-3C, 3ZSJ (58); galectin-10, 6L67 (59); pH6 antigen, 4F8L (60); and cholera toxin, 2CHB (61). The protein backbone is shown as a *cartoon* and the surface is highlighted. Protein and carbohydrate atoms in the binding site are shown as *sticks*. (*Bottom*) CH–π stacking interactions formed between β-D-galactose and the aromatic amino acid side chain are shown for each protein, with the protein backbone of the pocket shown as a cartoon. Individual CH–π interactions are shown as *yellow dotted lines*. Each of the proteins is shown and colored from *left* to *right*, as follows: progenitor toxin (*pink*), galectin-3C (*purple*), galectin-10 (*blue*), pH6 antigen (*green*), and cholera toxin (*yellow*). Carbohydrate carbon atoms are colored *black*, protein carbon atoms are colored as described above, all other atoms are colored as follows: oxygen in *red*, nitrogen in *blue*, and hydrogen in *white*. PDB, Protein Data Bank.

To understand the role of CH–π interactions in protein binding, we selected five proteins from our dataset that bind to monosaccharide β-D-galactose (galactose) with different CH–π stacking interaction orientations: progenitor toxin (57), galectin-3 carbohydrate recognition domain (galectin-3C) (58), galectin-10 (59), pH6 antigen (60), and cholera-toxin B-pentamer (61) (cholera toxin) (Fig. 2 and Supporting Information Figures S1–S5). Each of these five proteins forms a CH–π stacking interaction to the galactose monomer with a distinct orientation (Fig. 2). Progenitor toxin, galectin-3C, and cholera toxin all form CH–π interactions with carbon atoms 3, 4, 5, and 6, while the galectin-10 and pH6 antigen stacking interactions involve carbon atoms 1, 3, and 5. Even within these two groups, the angle of interaction and relative rotation of the rings differ among proteins (Fig. 2).

Our prior work demonstrated that this variation in CH–π stacking interaction orientation is representative of noncovalent protein–galactose binding sites in the PDB (39). We found that protein–galactose CH–π stacking interactions have a broad orientational landscape with many energetic minima that can be defined using two CVs that capture which C–H groups interact with the aromatic ring (39). The CVs are sums of the *d*_Cn-Ctr_ values described in Galactose CH–π interactions in protein–carbohydrate binding sites, where CV 1 is *d*_C1-Ctr_ + *d*_C2-Ctr_ and CV 2 is *d*_C4-Ctr_ + *d*_C6-Ctr_. We used these two CVs to perform WT-MetaD simulations on all five protein–galactose complexes to evaluate the free energy landscape of the binding interactions (see Experimental Procedures). The WT-MetaD simulations were run for at least 150 ns and until the carbohydrate dissociated from the binding pocket. By biasing along CVs that define the CH–π stacking interaction, we use these WT-MetaD simulations to understand the binding dynamics relative to the orientation of the CH–π stacking interaction. Importantly, while the MD force fields used to simulate these systems capture the van der Waals and electrostatic energies, the primary energetic drivers of CH−π interactions (39, 62), they do not specifically capture interactions between C−H groups and π orbitals (56, 63, 64). Thus, this analysis does not specifically assess CH−π interactions, but rather, more generally evaluates carbohydrate–aromatic interactions; however, for brevity, we will refer to the carbohydrate–aromatic stacking interactions modeled herein as CH−π stacking interactions.

**Figure 3:**
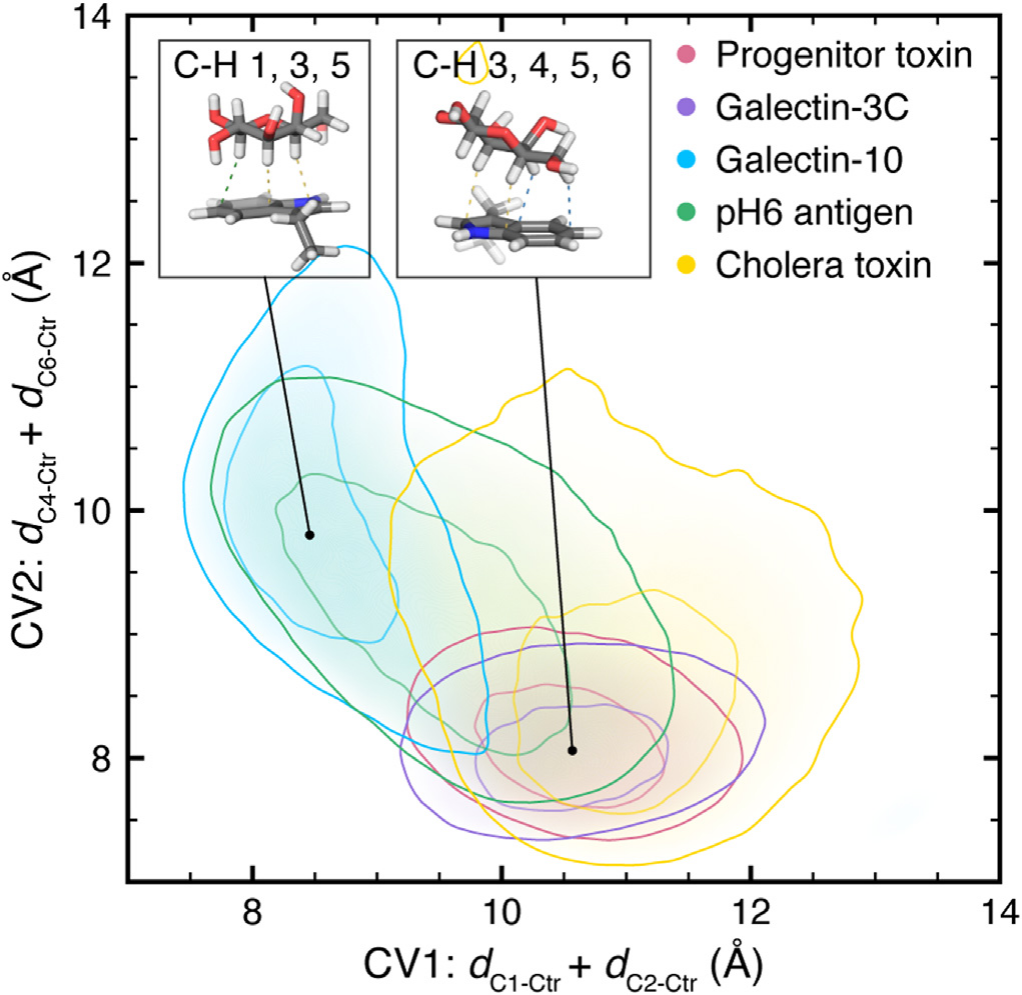
**Free energy landscapes of protein–β-D-galactose complexes.** WT-MetaD free energy landscapes of protein-monosaccharide complexes were plotted using the two collective variables that define the CH–π stacking interaction orientation: *d*_C1-Ctr_ + *d*_C2-Ctr_ (CV1) and *d*_C4-Ctr_ + *d*_C6-Ctr_ (CV2). For each complex, the contiguous region within 1.5 kcal/mol of the most favorable orientation is shown with contour lines at +0.5 and + 1.5 kcal/mol colored as defined in the inset key. Two inset images show example CH–π stacking interactions demonstrating orientations with (*upper left*) low CV1 values, which correspond to CH–π interactions formed by C–H groups on carbons 1, 3, and 5 or (*lower right*) low CV2 values, which correspond to CH–π interactions formed by C–H groups on carbons 3, 4, 5, and 6. Atoms are colored as follows: carbon in *gray*, nitrogen in *blue*, oxygen in *red*, and hydrogen in *white*. CV, collective variable; WT-MetaD, well-tempered metadynamics.

The WT-MetaD simulations of the five protein–galactose complexes have shallow free energy landscapes with binding free energies of ΔG = −3.2 to −5.7 kcal/mol (Fig. 3 and Supporting Information Figs. S6 and S7). The minimum free energy binding orientations of the five complexes do not converge to a singular CH−π stacking interaction orientation; rather, the proteins favor different orientations, as observed in their structures determined by X-ray crystallography. The galactose ligands also occupy different ranges of CH−π stacking interaction orientations in the simulations of each protein (Fig. 3 and Supporting Information Figs. S6 and S7). For example, while progenitor toxin and galectin-3C have similar free energy minima and local free energy landscapes, galectin-3C can form an alternative CH−π stacking interaction orientation with the 1, 3, and 5 C–H groups (CV1 = 8.8 Å, CV2 = 9.5 Å). This orientation is more favorable for galectin-3C than the progenitor toxin, because the galectin-3C protein binding pocket can accommodate more hydrogen bonds in that position (Supporting Information Fig. S6). On the other hand, galectin-10 strongly favors a CH−π stacking interaction orientation involving the 1, 3, and 5 C–H groups of galactose. The pH6 antigen shows no energetic preference between the binding orientation corresponding to a CH−π stacking interaction formed by either the 3, 4, 5, and 6, or the 1, 3, and 5 C–H groups (Fig. 3). These different preferences can be accounted for by considering the hydrogen bonds formed in each orientation. The pH6 antigen maintains the same hydrogen bonds regardless of which C–H groups participate in the CH−π stacking interaction orientation (Supporting Information Fig. S8). In the other protein–glycan complexes, shifting the angle of the CH−π stacking interaction disrupts hydrogen bonds. Thus, the position of hydrogen bonds in the binding pocket informs the orientational preferences of the CH−π stacking interactions in protein–carbohydrate binding sites.

Cholera toxin has the weakest binding interaction and explores the broadest range of CH−π stacking interaction orientations in simulations. The binding site is composed of flexible loops, which dynamically rearrange when bound to the monosaccharide galactose. In concert with this loop movement, the monomeric galactose ligand shifts, forming hydrogen bonds to different subsets of protein residues on the outer loops (*i.e.*, Glu51, Gln61, Asn90, and Lys91) or deeper into the binding pocket (*i.e.*, Glu51, His57, Gln61, Leu134, and Gly136) (Supporting Information Fig. S9). In both positions, galactose forms multiple hydrogen bonds with two or more amino acids, at least one of which is charged (Glu51 or Lys91), enabling binding in this range of orientations (Supporting Information Fig. S9). In contrast, the other four proteins have more structured galactose binding pockets; galactose interacts with protein residues primarily on regions with defined secondary structure. Interestingly, each of these four proteins has a monosaccharide or disaccharide glycan determinant (57, 60, 65, 66), while cholera toxin has a pentasaccharide glycan determinant (67), suggesting that more rigid binding pockets can enable protein recognition with shorter carbohydrate ligands.

**Figure 4:**
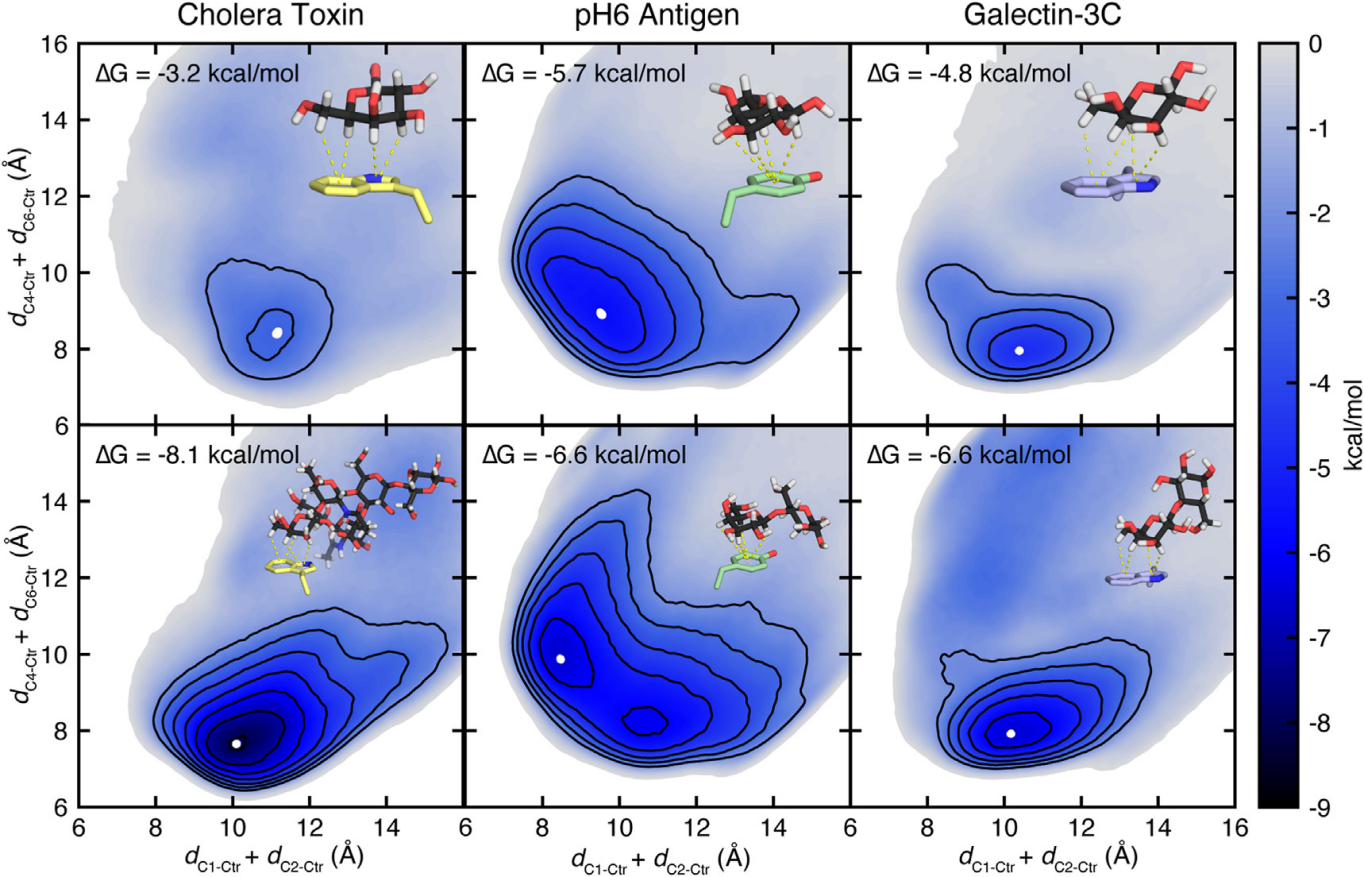
**Comparison of free energy landscapes of protein complexes with monosaccharide and oligosaccharide ligands.** Free energy landscapes of cholera toxin, pH6 antigen, and galectin-3C bound to (*top*) a galactose monomer and (*bottom*) an oligosaccharide ligand. All free energy landscapes are reported relative to two collective variables that define the CH–π stacking interaction orientation: *d*_C1-Ctr_ + *d*_C2-Ctr_ (CV1) and *d*_C4-Ctr_ + *d*_C6-Ctr_ (CV2). Energy landscapes are colored by binding free energy according to the inset color bar. Contour lines that encompass the minimum energy orientation are drawn in *black* in 1 kcal/mol increments starting at −2 kcal/mol, and the *white dot* represents the minimum energy orientation. For each system, the aromatic amino acid, galactose, and full carbohydrate ligand are visualized. The individual CH–π interactions are shown as *yellow dashed lines*. Atoms are colored as follows: protein carbon atoms in *yellow* (cholera toxin), *green* (pH6 antigen), and *purple* (galectin-3C), carbohydrate carbon atoms in *black*, oxygen in *red*, nitrogen in *blue*, and hydrogen in *white*.

Three of the proteins, galectin-3C, pH6 antigen, and cholera toxin, have been cocrystallized with oligosaccharides that have a terminal galactose residue in the position described above for monosaccharide binding (PDB IDs: 3ZSJ (58), 4F8O (60), and 2CHB (61)). In these structures, galectin-3C and pH6 antigen are bound to lactose, while cholera toxin is bound to the GM1 pentasaccharide. We performed WT-MetaD simulations on these complexes using the same CVs that define the CH−π stacking interaction of the terminal galactose (Fig. 4). The simulations were run until the galactose residue dissociated from its portion of the binding pocket. The results were used to assess the impact of additional carbohydrate residues on binding. The difference in free energies of the monosaccharide and oligosaccharide binding interactions varied across the three proteins in accordance with the bound oligosaccharide length. Cholera toxin binding to GM1 pentasaccharide is at least 4.9 kcal/mol more favorable than to galactose (1.2 kcal/mol per additional carbohydrate monomer), while for galectin-3C and pH6 antigen binding to lactose, the addition of a glucose residue (*i.e.*, a lactose ligand) contributed only an additional 1.8 and 0.9 kcal/mol, respectively (Fig. 4). Importantly, in galectin-3C and pH6 antigen, the anchoring galactose monomer contributes greater stabilization than the added glucose residue on lactose. Yet, for galectin-3C, lactose is a glycan determinant, suggesting that the free energy contribution of the additional carbohydrate residue plays a critical role in binding, even though it participates in limited interactions with the protein binding site.

WT-MetaD simulations reveal that the orientation of the free energy minimum CH−π stacking interaction differs between the monomeric and oligomeric carbohydrate ligands in complex with both cholera toxin and pH6 antigen (Fig. 4). In cholera toxin, the additional carbohydrate ligands form a network of hydrogen bonds to the protein, engaging a larger binding pocket and stabilizing the flexible loops (Supporting Information Fig. S10). As a result, the bound oligosaccharide ligand has one narrow free energy minimum orientation that maximizes all noncovalent interactions to the carbohydrate ligand. Galectin-3C also forms new hydrogen bonds between the added glucose residue and nearby residues Arg162 and Glu184, further stabilizing the minimum free energy binding orientation of the monosaccharide and decreasing the stability of the alternate CH–π stacking interaction formed by the 1, 3, and 5 C–H groups that was observed in the monomer (Supporting Information Fig. S11). The pH6 antigen forms only one additional hydrogen bond to Asp74, and as a result, the binding energy is within 1 kcal/mol of the monosaccharide binding interaction. However, this added hydrogen bond stabilizes the lactose orientation that involves a CH−π stacking interaction formed by the 1, 3, and 5 C–H groups, and to a lesser extent, the CH–π stacking interaction formed by the 3, 4, 5, and 6 C–H groups, resulting in a more specific orientational preference as compared to the monosaccharide binding interaction (Supporting Information Fig. S12).

We ran 200-ns unbiased MD simulations starting from the structures determined by X-ray crystallography. Snapshots from these MD runs were analyzed with molecular mechanics with generalized Born surface area (MM/GBSA) energy decomposition analysis. MM/GBSA offers an enthalpic energy estimation of all binding interactions that can be decomposed into per-residue contributions and their electrostatic, van der Waals, and solvation energy components (Table 1 and Supporting Information Tables S2 and S3). The total MM/GBSA energies were correlated to the WT-MetaD free energies, R^2^ = 0.85, but the neglect of entropic penalties leads to the MM/GBSA binding energies being much higher than the WT-MetaD free energies (Supporting Information Fig. S13). Decomposing the MM/GBSA total energies into per-residue contributions shows that the aromatic amino acids that form CH–π stacking interactions (Trp or Tyr) with the galactose have a significant enthalpy contribution (−2.4 to −3.5 kcal/mol, Table 1). In most cases, the CH–π energy is dominated by the van der Waals contribution (−2.2 to −2.9 kcal/mol) with weaker electrostatic and solvation energies (Table 1). Compared to amino acids that form hydrogen bonds and have highly favorable electrostatic energies that are largely offset by unfavorable solvation energies (R^2^ = −0.96), the aromatic amino acids that form CH–π interactions can confer a greater total energy contribution due to the limited solvation energy offset (Supporting Information Tables S4–S8). Thus, in each of the five proteins, the aromatic amino acids that form CH–π stacking interactions rank among the top three amino acid residues in total binding energy contribution (Supporting Information Tables S4–S8).

**Table 1:** MM/GBSA total interaction energies and enthalpy contributions from the aromatic amino acid (Ar AA) that forms the CH–π stacking interaction (tryptophan or tyrosine)

Protein	Monosaccharides					Oligosaccharides				
	Total energy	Ar. AA total energy	Ar. AA vdW	Ar. AA Elst.	Ar. AA Solv.	Total energy	Ar. AA total energy	Ar. AA vdW	Ar. AA Elst.	Ar. AA Solv.
Galectin-3C	−14.0	−3.0	−2.6	−0.9	0.5	−26.4	−3.2	−2.9	−0.7	0.4
Cholera toxin	−10.0	−2.4	−2.2	−0.8	0.6	−31.2	−4.1	−3.2	−3.5	2.7
pH6 Antigen	−19.9	−2.4	−2.5	−0.9	1.0	−21.5	−2.9	−3.4	−1.2	1.7
Progenitor toxin	−20.8	−3.5	−2.8	−0.9	0.2	-	-	-	-	-
Galectin-10	−17.1	−3.5	−2.9	−0.4	−0.2	-	-	-	-	-

Energies are evaluated for complexes formed by galectin-3C, cholera toxin, pH6 antigen, progenitor toxin, and galectin-10 with monosaccharide galactose. Energies are also reported for oligosaccharide complexes: galectin-3C with lactose (Gal(β1-4)Glc), cholera toxin with GM1 pentasaccharide (Gal(β1-3)GalNAc(β1-4)[Neu5Ac(α2-3)]Gal(β1-4)Glc), and pH6 antigen with lactose (Gal(β1-4)Glc). Aromatic amino acid enthalpy contributions are broken down into van der Waals (vdW), electrostatics (Elst), and solvation (Solv.: Generalized Born [GB] + Solvent Accessible Surface Area [SASA]) energies. All energies are reported in kcal/mol.

Comparing the MM/GBSA results for the protein complexes with monosaccharide *versus* oligosaccharide ligands, we note that the oligosaccharide complexes had more favorable enthalpy contributions from the aromatic amino acid than their monosaccharide counterparts, even though the additional carbohydrate residues on the oligosaccharide did not directly interact with the aromatic residue (Table 1). Other residues that similarly interact only with the galactose have comparable increases in enthalpy contributions in the oligosaccharide complexes. Thus, the orientational stabilization provided by additional carbohydrate residues on the oligosaccharide results in more favorable enthalpies for all contributing interactions. These findings indicate that multiple carbohydrate residues on an oligosaccharide ligand can contribute by stabilizing one binding orientation and increasing the enthalpic favorability of the existing interactions.

### Hydrogen bonds influence the orientation of CH−π interactions

Given that the formation of additional hydrogen bonds influences the orientation of the CH–π stacking interactions in oligosaccharides, we set out to determine how ablation of key hydrogen bonds in a protein binding pocket would influence the CH–π stacking interaction orientation. We analyzed galectin-3C due to the available experimental binding affinity data on relevant side-chain substitutions (26).

**Figure 5:**
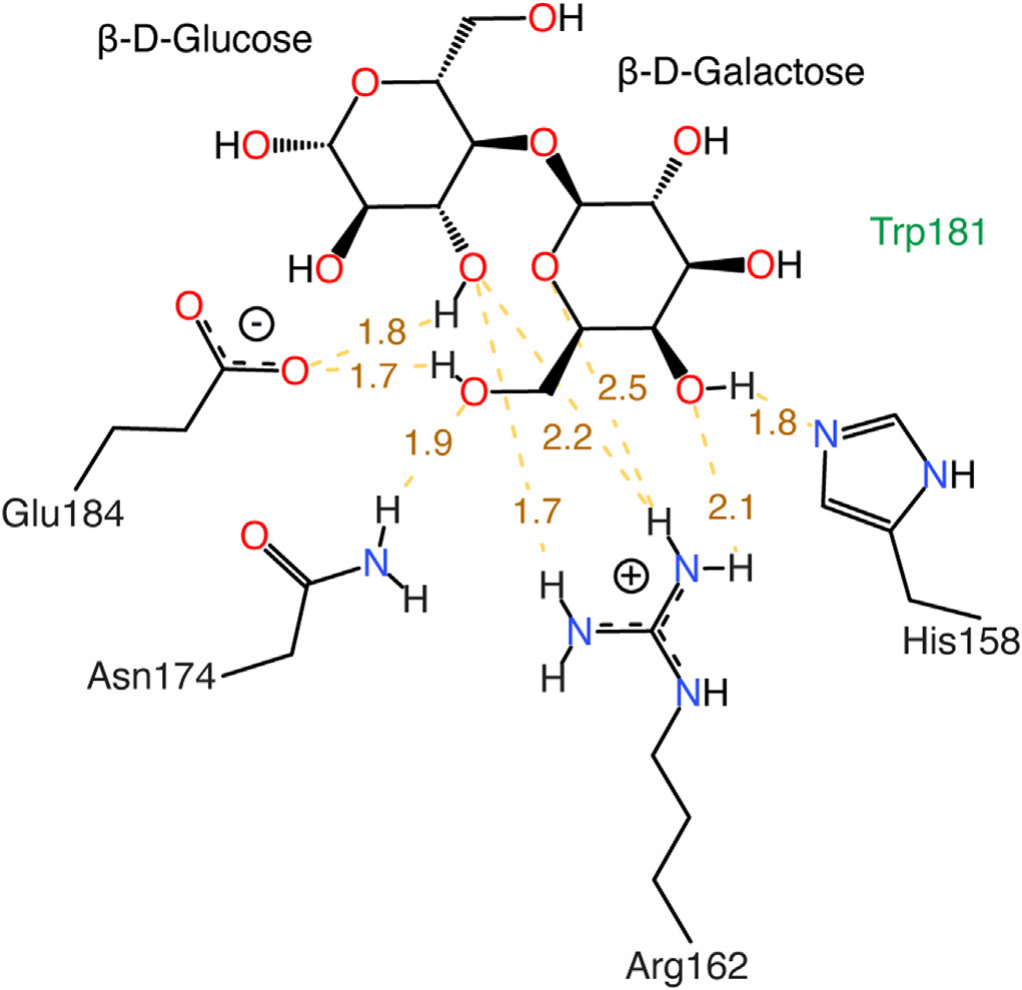
**Visualization of the WT galectin-3C binding pocket.** Interacting residues Glu184, Asn174, Arg162, and His158 are shown. Trp181 is noted by a *green label*. Hydrogen bonds are shown as *yellow dotted lines*, and their distances are labeled in Å.

**Figure 6:**
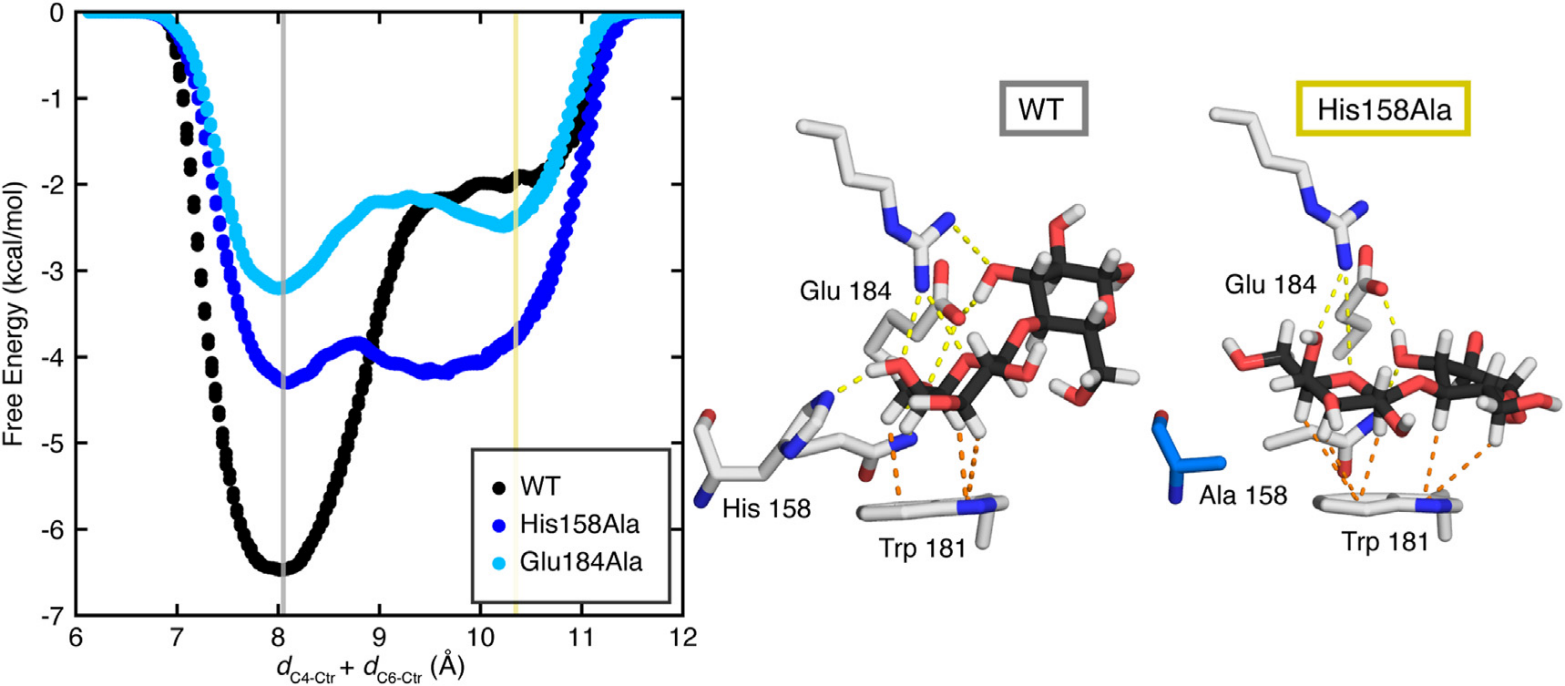
**Comparison of free energy surfaces and binding orientations of the WT and variant Gal3C–lactose complexes.** (*Left*) Free energy surfaces computed from WT-MetaD simulations of wildtype (WT, *black*), His158Ala (*light blue*), and Glu184Ala (*dark blue*) galectin-3C. Using the two collective variables, *d*_C1-Ctr_ + *d*_C2-Ctr_ (CV1) and *d*_C4-Ctr_ + *d*_C6-Ctr_ (CV2), free energies of all three complexes were evaluated along the line CV2 = −1.1∗CV1 +19.4 to capture both energetic minima and plotted relative to the corresponding value of CV2 (*d*_C4-Ctr_ + *d*_C6-Ctr_) in Å. The free energy minimum for all three complexes is indicated with a *gray line*. The second free energy minima, which is observed only for the two mutant complexes, is marked with a *yellow line*. (*center* and *right*) The binding interactions for both free energy minima are shown. (*center*) The free energy minimum with CV2 = 8.05 (*gray line*) is shown for the WT complex and (*right*) the free energy minimum with CV2 = 10.35 (*yellow line*) is shown for the His158Ala mutant. Atoms are colored as follows: oxygen in *red*, hydrogen in *white*, nitrogen in *dark blue*, carbohydrate carbon atoms in *black*, protein carbon atoms in *gray*, and mutant residue 158 carbon atoms in *light blue*. CH−π interactions shown as *orange dotted lines* and hydrogen bonds shown as *yellow dotted lines*. WT-MetaD, well-tempered metadynamics.

In a galectin-3C complex, the lactose ligand forms a CH–π stacking interaction with Trp181 and hydrogen bonding interactions to four amino acids, His158, Arg162, Asn174, and Glu184 (Fig. 5). We tested the effect of disrupting hydrogen bonds by creating two mutations, His158Ala and Glu184Ala. The His158Ala mutation results in the loss of one hydrogen bond to the glucose, while the Glu184Ala mutation results in the loss of two charged hydrogen bonds to the glucose and galactose (Fig. 5). We performed WT-MetaD simulations using the same CVs until the lactose ligand dissociated entirely from the binding pocket. We used these simulations to assess the impact of the loss of hydrogen bonds on the binding free energy with respect to the CH−π stacking orientation. Both mutant proteins have weaker free energies of binding to lactose than the WT protein by at least 2 kcal/mol, −4.6 and −3.2 kcal/mol for His158Ala and Glu184Ala, respectively (Fig. 6). When these results are compared to prior experimental studies, both approaches show significant decreases in binding affinity (WT, His158Ala, and Glu184Ala experimental ΔG = −5.4, −1.7, and −1.5 kcal/mol, respectively), demonstrating that the WT-MetaD results capture similar energetics to the experimental analysis (26).

In evaluating the free energy landscapes of binding, we observed that both mutant proteins occupy an alternative binding orientation. This second minimum-energy conformation is stabilized by a CH–π stacking interaction involving the 1, 3, and 5 C–H groups of galactose and the 4 and 6 C–H groups of glucose, rather than the 3, 4, 5, and 6 C–H groups of galactose, as in the optimal binding orientation (Fig. 6). This alternate orientation is similar to the one occupied by monosaccharide galactose when bound to galectin-3C *via* C–H groups 1, 3, and 5 (Fig. 4). Thus, the disruption of hydrogen bonds results in greater orientational variability of the bound carbohydrate ligand.

### Orientational flexibility of CH−π interactions enables processivity

**Figure 7:**
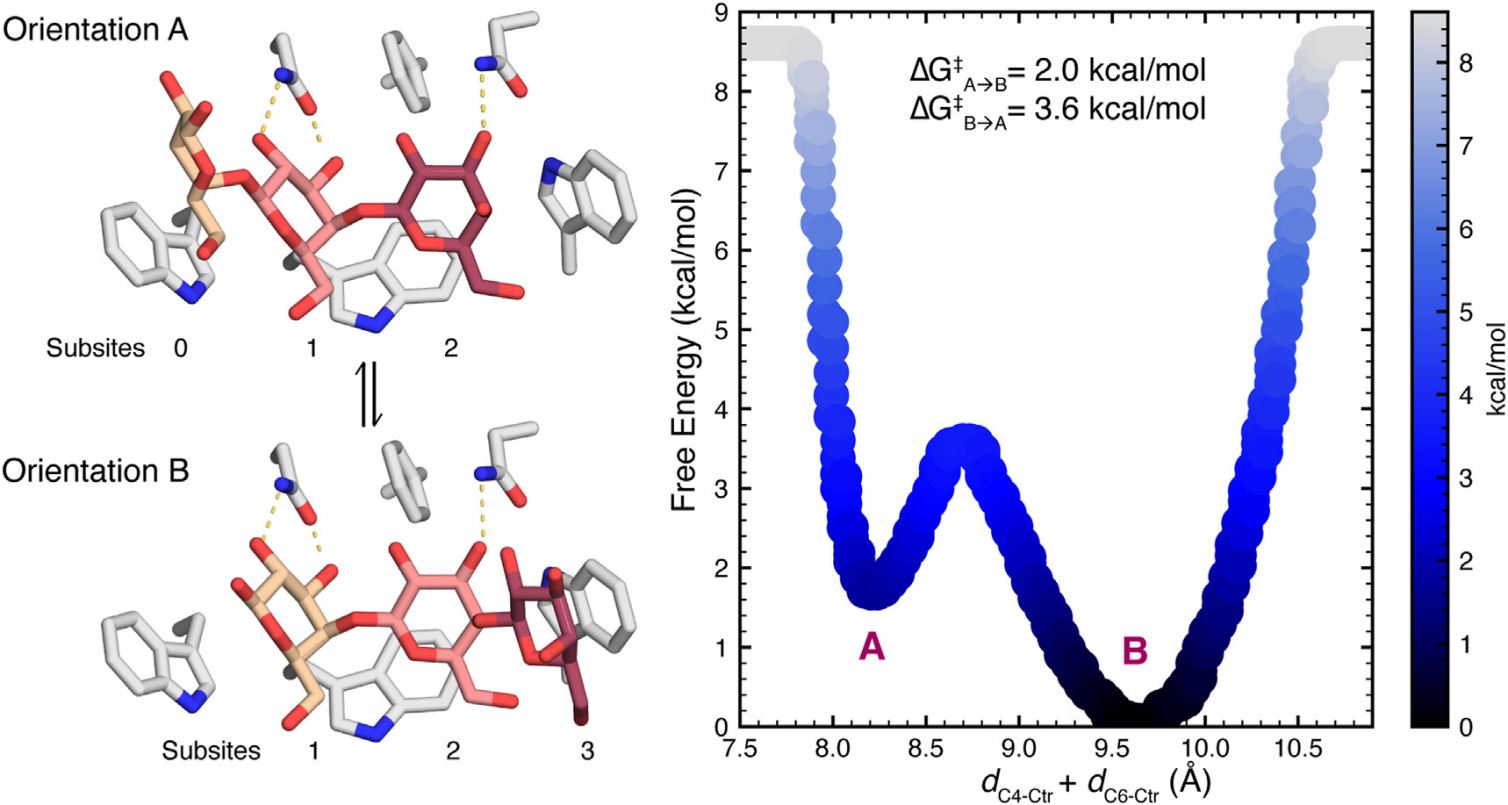
**Translocation of galactotriose in TmCBM61.** (*Left*) Visualization of the binding interactions of orientations *A* and *B* (PDB ID: (*A*) 2XON and (*B*) 2XOM) of galactotriose bound to TmCBM61. Protein atoms are colored as follows: carbon in *gray*, nitrogen in *blue*, and oxygen in *red*. Carbohydrate oxygen atoms are colored in *red*, and carbon atoms are colored by position in the chain, from *left* to *right*, wheat, salmon, and raspberry. Hydrogen bonds are shown as *dotted yellow lines*. (*right*) Free energy surface computed from WT-MetaD simulations of the TmCBM61-galactotriose complex with collective variables *d*_C1-Ctr_ + *d*_C2-Ctr_ (CV1) and *d*_C4-Ctr_ + *d*_C6-Ctr_ (CV2). Free energies along the line CV2 = −0.4∗CV1 +13.4 are plotted relative to the value of CV2 (*d*_C4-Ctr_ + *d*_C6-Ctr_) in Å. Points are colored by free energy and reported in kcal/mol. Optimal orientations of the complex in A and B are shown on the plot, and the free energy barriers to translocation are reported for both directions. PDB, Protein Data Bank; TmCBM61, carbohydrate-binding module 61 of *Thermotoga maritima*; WT-MetaD, well-tempered metadynamics.

Having observed that CH–π stacking interactions can stabilize multiple binding orientations, we next explored the dynamics of protein–oligosaccharide binding sites with multiple aromatic residues that can engage in CH–π stacking interactions. We selected one such protein, carbohydrate-binding module (CBM) 61 of *Thermotoga maritima*, TmCBM61, bound to a galactotriose ligand (PDB IDs: 2XON and 2XOM). TmCBM61 is a bacterial protein subunit commonly associated with hydrolases and is believed to play a key role in plant cell wall depolymerization (68). TmCBM61 can bind a helical β-1-4-galactan structure *via* three contiguous tryptophan residues, each of which forms CH–π stacking interactions with galactose residues. TmCBM61 has been crystallized in complex with a galactotriose ligand in two unique and overlapping binding orientations, A and B (Fig. 7). In orientation A, binding subsites 0, 1, and 2 are occupied by the galactotriose, while in orientation B, binding subsites 1, 2, and 3 are occupied, and in both orientations, the galactose residues in subsites 1 and 2 maintain the same relative positions (Fig. 7). Given the role of this CBM in processive depolymerization of β-1-4-galactans, we aimed to evaluate the energy barrier to translocation between these orientations (68).

Starting with TmCBM61 bound to galactotriose in orientation A (PDB ID: 2XON), we performed WT-MetaD simulations to elucidate the free energy landscape of the bound complex relative to the CH–π stacking interactions (Fig. 7). Given that this complex involves three CH–π stacking interactions, we biased the WT-MetaD simulations using the prior collective variables (CV1: *d*_C1-Ctr_ + *d*_C2-Ctr_ and CV2: *d*_C4-Ctr_ + *d*_C6-Ctr_) computed on the central galactose and central tryptophan (Trp154) residues. These simulations showed that both orientations of the TmCBM61–galactotriose complex are energetically favorable, yet orientation B, which is primarily stacked above Trp154 and Trp123, is more favorable (Fig. 7). Thus, the galactose binding to subsite 3 is more favorable than to subsite 0.

Notably, the free energy barrier to translocation of the galactotriose ligand from orientation A into orientation B is only 2 kcal/mol, while in the reverse direction it is 3.6 kcal/mol (Fig. 7). As such, bidirectional translocation is readily accessible at room temperature, but the translocation from orientation A into orientation B is faster. Yet, in each case, the approximate transition state (*i.e.*, the highest-energy structure along the free energy path) occupied during ligand translocation is considerably more favorable than dissociation of the ligand. These data suggest that the ligands can “slide” along the binding site, a process that would facilitate catalytic hydrolysis of the oligosaccharide.

We next verified these observations with more rigorous quantum mechanical (QM) calculations of the potential energy surface (*i.e.*, neglecting computationally demanding entropic corrections) for translocation (Supporting Information Texts S1 and S2 and Table S9). This analysis revealed that the main contributor to energy barriers for translocation is the breaking of hydrogen bonds in the active site (Supporting Information Tables S9–10 and Fig. S14). If we remove the hydrogen bond partners (*i.e.*, Asn57 and Asn66) from the QM cluster model, we observe lower energy barriers for translocation, with the highest-energy transition state decreasing in energy from 8.2 kcal/mol to 3.7 kcal/mol (Supporting Information Table S11 and Figs. S15–S17). Thus, hydrogen bonds stabilize specific binding conformations, while CH–π stacking interactions support translocation (Fig. 7 and Supporting Information Figs. S15–S17). The orientational flexibility of CH–π stacking interactions, which can stabilize multiple distinct binding orientations, facilitates translocation by maintaining favorable interactions. In this case, a binding site with three contiguous CH–π stacking interactions and only three additional hydrogen bonds results in two highly favorable binding orientations for galactotriose and a low barrier to translocation between them. By stabilizing binding during ligand transitions, CH–π stacking interactions limit ligand dissociation and increase the rate of translocation. Thus, the formation of multiple CH–π stacking interactions plays a key role in supporting the translocation necessary for processive enzyme activity in TmCBM61.

## Discussion

CH–π stacking interactions are pervasive in noncovalent protein–carbohydrate binding sites (6). Prior experimental and computational studies of glycan–aromatic interactions have shown that CH–π stacking interactions formed by β-D-galactose are highly favorable and have a broad orientational landscape (39, 42, 53). However, there was limited knowledge of the influence of the protein environment on CH−π stacking interaction orientational dynamics. In addition, the functional interplay of hydrogen bonds and CH–π stacking interactions in binding was unclear.

Thus, we studied several complexes that form one CH–π stacking interaction to a β-D-galactose moiety: progenitor toxin, galectin-3C, galectin-10, pH6 antigen, or cholera toxin bound to a monosaccharide, disaccharide, or pentasaccharide ligand. We performed WT-MetaD simulations using CVs that define the orientation of the CH–π stacking interaction to obtain binding free energy landscapes of these protein–carbohydrate interactions and determine the influence of the protein environment on the CH−π stacking interaction orientational dynamics. All protein–monosaccharide complexes occupied a range of CH–π stacking interactions, yet each favored a distinct orientation, demonstrating that the observed orientational variance under crystalline conditions is maintained in the solution state. Complexes with flexible binding sites, which we define as those in which the galactose formed similar numbers of hydrogen bonds in multiple different binding orientations, explored more CH–π stacking interaction orientations. Thus, hydrogen bonds play a key role in defining the binding orientation.

To determine the effect of increasing the number of hydrogen bonds on binding affinity and orientational flexibility, we studied extended carbohydrate ligands in protein–oligosaccharide complexes. In all cases, binding to extended oligosaccharide ligands resulted in more specific orientational dependence, higher binding affinity, and a higher enthalpy contribution than binding to β-D-galactose. This finding is consistent with experimental data that indicate that most glycan-binding proteins have higher affinity interactions with oligosaccharides composed of 2 to 6 residues than monosaccharides (7, 8). Our data is also complementary to prior observations that show that even carbohydrate residues that form limited interactions with the protein, such as the glucose residue of the oligosaccharide bound to pH6 antigen, contribute an increased binding affinity due to the nonbinding units perturbing solvation shells and increasing the entropy (8, 69).

To test the role of hydrogen bonds in influencing CH–π stacking interaction orientation, we removed hydrogen-bonding side chains in galectin-3C and analyzed the resulting complexes with lactose. These replacements not only lowered the binding affinity, as previously noted (26), but also broadened the free energy landscape significantly. Therefore, hydrogen bonds play an essential role in constraining the binding orientation, and their disruption results in greater orientational variability of a bound carbohydrate ligand. Because CH–π stacking interactions allow a broad range of orientations, optimal binding stability is achieved only when the available hydrogen bonds are also satisfied. Thus, hydrogen bond positioning in the binding pocket drives the orientation of CH–π stacking interactions.

Although our simulations provide a comprehensive view of CH–π stacking interaction dynamics, we acknowledge limitations inherent to the computational methods. The MD force fields do not specifically evaluate the CH–π interaction energy, but rather, they capture general van der Waals and electrostatic contributions. In addition, the WT-MetaD and MM/GBSA methods, while powerful for sampling conformational landscapes and decomposing enthalpic contributions, respectively, do not fully capture the complexity of binding in cellular environments in either case, and entropic effects in the latter case. Future studies that integrate experimental validation—such as NMR measurements, calorimetry, or site-directed mutagenesis—could further test the predicted binding orientations and energetic contributions observed here.

**Figure 8:**
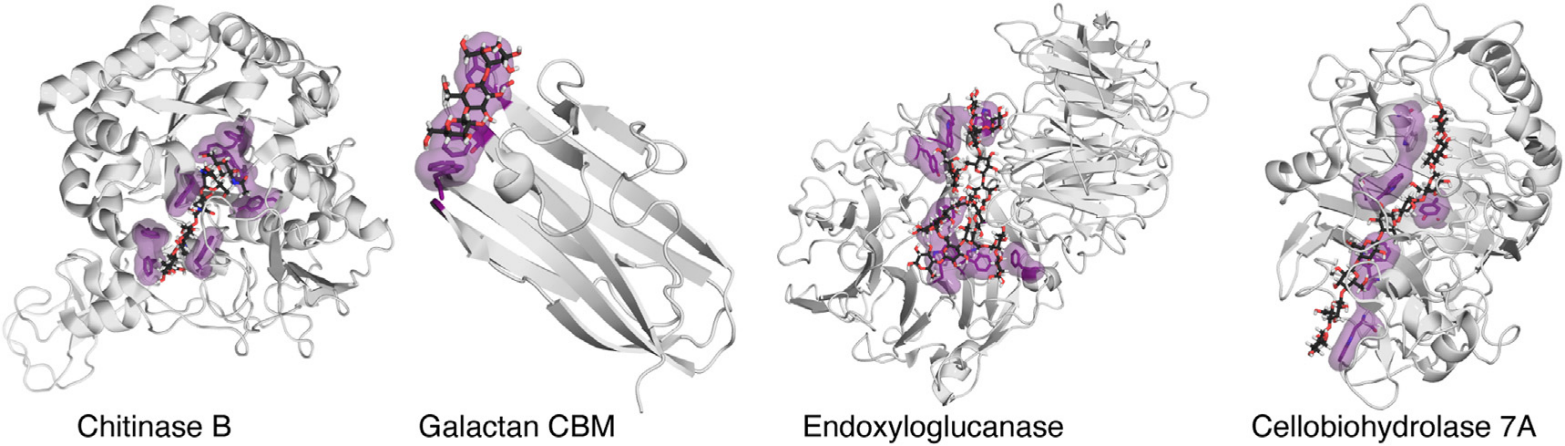
**Examples of enzymes that form multiple CH–π stacking interactions with oligosaccharide ligands.** Visualization of TmCBM61 and a selection of enzymes that form multiple CH−π stacking interactions to complex carbohydrate ligands (PDB IDs: 1E6N (70), chitinase B; 2XON (68), TmCBM61; 4C4C (71), cellobiohydrolase 7A; 5FKS (72), endo-xyloglucanase). Protein–carbohydrate complexes are shown with the protein backbone drawn as a *gray* cartoon, the carbohydrate ligands are shown as *sticks* and the aromatic amino acids involved in CH−π stacking interactions are colored in *purple* and represented as *sticks* with the surface shown. *Stick* representations of atoms are colored as follows: aromatic carbon in *purple*, carbohydrate carbon in *black*, oxygen in *red*, nitrogen in *blue*, and hydrogen in *white*. PDB, Protein Data Bank; TmCBM61, carbohydrate-binding module 61 of *Thermotoga maritima*.

The orientational flexibility of CH–π stacking interactions also has functional consequences in enzymes. Enzymes, especially the hydrolases responsible for carbohydrate degradation, are enriched for the formation of multiple CH–π stacking interactions (PDB IDs: 1E6N (70), chitinase B; 4C4C (71), cellobiohydrolase 7A; 5FKS (72), endo-xyloglucanase, Fig. 8). Prior studies have shown that the tryptophan residues in these enzymes are essential for ligand recognition (cellobiohydrolase and chitinase B) (44, 45, 73, 74), can result in greater reactivity (chitinase B) (46), and can contribute to processive action (endo-xyloglucanase) (45, 47, 75). Our WT-MetaD simulations and QM cluster models of TmCBM61 extend these findings, demonstrating that CH–π stacking interactions lower energetic barriers to translocation. The formation of multiple CH–π stacking interactions results in a broader free energy landscape of binding as each CH–π stacking interaction confers some local orientational flexibility, resulting in multiple favorable orientations that can maintain ligand binding. We hypothesize that the contiguous positioning of these interactions that we observe in enzymes serves to lower the free energy barrier to translocation, enhancing processive enzyme activity. This functional role of the broad orientational landscape of CH–π stacking interactions may serve as a blueprint for designing molecular processes to act on polymers of all kinds.

## Conclusion

CH–π stacking interactions contribute significantly to glycan binding, allowing a range of orientations. The greater orientational flexibility of CH–π stacking interactions works in concert with more directional hydrogen bonds to drive essential biological functions from recognition to translocation. The relative number and position of hydrogen bonds and CH–π stacking interactions in a protein binding site determine the balance of effects that govern ligand orientation and movement. Tuning these properties in a protein–carbohydrate binding site should enable selective recognition of one or more carbohydrate ligands. Side chain substitution can also modulate enzyme activity—either enhancing or inhibiting function—toward specific ligands. These insights offer a framework for engineering protein systems with selective carbohydrate recognition, understanding the consequences of different mutations on glycan-binding protein function, and designing enzymes that process a broad range of biological or synthetic oligosaccharides.

## Experimental procedures

To model the energetic landscapes of the protein–carbohydrate complexes, we first prepared the structures for simulations. We obtained 3D coordinates for all proteins from the PDB (54). For galectin-3C and cholera toxin, we also truncated carbohydrate ligands to produce the monosaccharide–protein complexes. Protonation states were assigned using the H++ web server (76) with an internal dielectric constant of 10.0, a pH of 7.0, and all other default settings applied. The residue labels for the ligands were manually updated to match the GLYCAM_06 (63) force field naming conventions. The AMBER tLEaP program (77) was used to solvate each protein in a water box with at least 10 Å initial buffer around all protein dimensions. tLEaP was then used to neutralize each system using either sodium or chloride ions. tLEaP was also used to generate final topology and coordinate files. We used forcefields ff14SB (64) for the protein, TIP3P (78) for water, and GLYCAM_06j-1 for carbohydrate ligands. Final system sizes and charges are listed in Supporting Information Table S12, and input files are available in the Zenodo (55) repository.

Using the resulting input structures, we ran MD and WT-MetaD simulations to assess the binding dynamics. Simulations were performed with AMBER18 (77) using the GPU-accelerated particle mesh Ewald (PME) (79) molecular dynamics (PMEMD) module (80). Each simulation was equilibrated using the following procedure. We ran a 2000-cycle minimization of the water molecules and hydrogen atoms with a restraint applied to protein and ligand heavy atoms. Next, we ran a 2000-cycle minimization of all atoms with a restraint applied to the heavy atoms of the galactose and aromatic amino acid involved in the CH−π interaction (*i.e.*, C, O, and N atoms). All restraints had a 200 kcal mol^-1^Å^-2^ weight. Next, we performed a 10 ps controlled NVT equilibration to heat the system from 100K to 300K and a 10 ps NpT equilibration using the Berendsen barostat with a pressure relaxation time of 1 ps. Both steps used a Langevin thermostat for temperature control with a collision frequency of 5.0 ps^-1^, a 2 fs timestep, periodic boundary conditions, the SHAKE algorithm (81) for constraining hydrogen atom bond lengths, and restraints on the heavy atoms of the CH−π interaction species. Finally, a 1 ns NpT equilibration was performed with the same parameters and no restraints to allow free equilibration of the CH-π interaction.

WT-MetaD simulations were performed using the open-source PLUMED library version 2.9 (31, 82, 83, 84) with the two CVs described in the main text. Simulations were run for at least 150 ns, and for monosaccharide complexes, until the galactose ligand had fully dissociated; for oligosaccharide complexes, until the galactose residue left the binding pocket; and for TmCBM61 until the translocation barrier was unchanging. Simulations were run at 300K with Gaussian hills deposited every 500 steps with σ values of 0.01 Å. Gaussian hill heights were set to 0.05 kJ/mol for WT oligosaccharide binding interactions and 0.025 kJ/mol for all other systems and employed with a bias factor of 20. Parameters were selected *via* trial-and-error assessments of convergence of the expected free energy profile (Supporting Information Figs. S18–S22). A 1.5 Å upper wall restraint was applied to both CVs, with an energy constant of 150 kJ/mol. The restraint was maintained for at least 150 ns, then released to enable ligand dissociation to occur after the bias filled the free energy well of binding. The full MD equilibration and WT-MetaD procedure was carried out three times for each system. Final energy landscapes are the averaged results of the three independent WT-MetaD simulations for each system.

Direct MD (*i.e.*, 200 ns) simulations were carried out to collect statistics for energy decomposition analysis at the MM level of theory. Each system was equilibrated and simulated three times and the resulting production simulations were combined using the AMBER CPPTRAJ trajectory processing program (85). Each concatenated trajectory was clustered using the k-means algorithm implemented in CPPTRAJ (85), with k = 5, to identify the major structural conformations of the equilibrated systems. The resulting trajectory of the predominant cluster was used as input for MM/GBSA (32) evaluation of binding interaction energetics. The MM/GBSA calculations were performed using the AMBER MMPBSA.py script (86) with the Onufriev, Bashford and Case (OBC) generalized Born (GB) model (87) and salt concentration set to 0.1. Total interaction energies are decomposed into pairwise per-residue energies for relevant residues with 1 to 4 terms added to internal potential terms (Supporting Information Table S13). Trajectory snapshots were spaced at least 20 ps apart, and the total number of snapshots used for each system is included in Supporting Information Table S14.

Cluster models suitable for QM modeling of the binding interaction between galactotriose and *Tm*CBM61 were constructed using 3D coordinates from PDB entries 2XON and 2XOM (Supporting Information Text S1). Density functional theory calculations were performed at the B3LYP (88, 89)/6-31G∗ level to optimize initial geometries, interpolate intermediate structures, and evaluate the minimum energy path using the climbing image nudged elastic band (CI-NEB) (90) method (Supporting Information Text S2).

## Data availability

Data are included in the article, supporting information, and linked on Zenodo at https://doi.org/10.5281/zenodo.15062118. Any questions regarding data availability can be directed to the corresponding authors.

## Supporting information

This article contains supporting information (88, 89, 90, 91, 92, 93, 94, 95, 96, 97, 98).

## Conflict of interest

The authors declare that they have no conflicts of interest with the contents of this article.
