# Supplementary material for: CH−π interactions confer orientational flexibility in protein–carbohydrate binding sites
Source: J Biol Chem. 2025 Jun 14;301(8):110379. doi: 10.1016/j.jbc.2025.110379 (PMC12309604; doi:10.1016/j.jbc.2025.110379)
Supplement: Supplemental data [file mmc1.pdf]

Supporting Information for

CH–π Interactions Confer Orientational Flexibility in Protein–Carbohydrate Binding Sites

Allison M. Keys[1,2,3], David W. Kastner[2,3,4], Laura L. Kiessling[3,5,6,*], and Heather J. Kulik[2,3,5]*

[1]Computational and Systems Biology Program, Massachusetts Institute of Technology, Cambridge, MA 02139, USA
[2]Department of Chemical Engineering, MIT, Cambridge, MA 02139, USA
[3]Department of Chemistry, MIT, Cambridge, MA, USA 02139, USA
[4]Department of Biological Engineering, MIT, Cambridge, MA, USA 02139, USA
[5]The Broad Institute of MIT and Harvard, Cambridge, MA 02142, USA
[6]Koch Institute for Integrative Cancer Research, MIT, Cambridge, MA 02142, USA
*co-corresponding authors: kiessling@mit.edu, hjkulik@mit.edu

Contents

Table S1 Proteins with multiple CH–π stacking interactions ... Page S3
Figure S1 Progenitor toxin binding pocket visualization ... Page S4
Figure S2 Galectin-3C binding pocket visualization ... Page S4
Figure S3 Galectin-10 binding pocket visualization ... Page S5
Figure S4 pH6 antigen binding pocket visualization ... Page S5
Figure S5 Cholera toxin binding pocket visualization ... Page S6
Figure S6 Monosaccharide free energy landscapes ... Page S6
Figure S7 Monosaccharide free energy landscapes versus crystal structures ... Page S7
Figure S8 Cholera toxin – galactose binding orientation comparison ... Page S7
Figure S9 pH6 antigen – galactose binding orientation comparison ... Page S8
Figure S10 Cholera toxin – GM1 pentasaccharide binding pocket visualization ... Page S8
Figure S11 pH6 antigen – lactose binding pocket visualization ... Page S9
Figure S12 Galectin-3C – lactose binding pocket visualization ... Page S9
Table S2 MM/GBSA monosaccharide energetics ... Page S10
Table S3 MM/GBSA oligosaccharide energetics ... Page S10
Figure S13 WT–MetaD versus MM/GBSA total energy comparisons ... Page S11
Table S4 Galectin-3C MM/GBSA residue decomposition energetics ... Page S11
Table S5 Cholera toxin MM/GBSA residue decomposition energetics ... Page S11
Table S6 pH6 antigen MM/GBSA residue decomposition energetics ... Page S12
Table S7 Progenitor toxin MM/GBSA residue decomposition energetics ... Page S12
Table S8 Galectin-10 MM/GBSA residue decomposition energetics ... Page S12
Text S1 QM cluster model preparation ... Page S13
Text S2 QM cluster model calculation ... Page S13
Table S9 Translocation QM cluster model contents ... Page S14
Table S10 Description of translocation steps ... Page S14

Figure S14 Visualization of reaction intermediates Page S15
Table S11 Tabulated energetics for intermediates and transition states Page S15
Figure S15 NEB potential energy surface with all residues Page S16
Figure S16 NEB potential energy surface with only tryptophan Page S16
Figure S17 Energy profile for translocation with and without protein residues Page S17
Table S12 Simulation Input information Page S18
Figure S18 Effect of metadynamics bias factor on energetic landscape Page S18
Figure S19 Effect of metadynamics hill height on energetic landscapes Page S19
Figure S20 WT–MetaD convergence with simulation progress - monosaccharide Page S19
Figure S21 WT–MetaD convergence with simulation progress - oligosaccharide Page S20
Figure S22 WT–MetaD convergence with simulation progress – TmCBM61 Page S21
Table S13 Amino acids involved in MM/GBSA decomposition Page S22
Table S14 Frames used for MM/GBSA calculations Page S22
References Page S23

Table S1. Table of all protein–carbohydrate complexes with two or more CH–π stacking interactions between residues on the protein and a carbohydrate ligand, including duplicates.

PDB ID	Classification	Protein Name	Carb. Length
5JM1	SUGAR BINDING PROTEIN	Agglutinin alpha chain	3
2EAD	HYDROLASE	Alpha-fucosidase	3
2XOM	HYDROLASE	Arabinogalactan endo-1-4-β galactosidase	3
2XON	HYDROLASE	Arabinogalactan endo-1-4-β galactosidase	3
2CN3	HYDROLASE	β-1,4-xyloglugan hydrolase	8
1URX	HYDROLASE	β-agarase A	4
4ATF	HYDROLASE	β-agarase B	8
2VU9	HYDROLASE	Botulinum neurotoxin A heavy chain	6
2YFZ	SUGAR BINDING PROTEIN	Carbohydrate binding family 6	5
3WKH	ISOMERASE	Cellobiose 2-epimerase	2
6UG7	IMMUNE SYSTEM	ch28/11 Fab light chain	6
6UG8	IMMUNE SYSTEM	ch28/11 Fab light chain	6
2WNF	TRANSFERASE	CMP-N-acetylneuraminate-β-galactosamide-α-2,3-sialyltransferase	2
2WNB	TRANSFERASE	CMP-N-acetylneuraminate-β-galactosamide-α-2,3-sialyltransferase	2
1V3M	TRANSFERASE	Cyclomaltodextrin glucanotransferase	2
5FKS	HYDROLASE	Endo-1,4-β-glucanase/xyloglucanase, G74A	7
5A58	HYDROLASE	Endo-α-galactosaminidase	2
5A56	HYDROLASE	Endo-α-acetylgalactosaminidase	2
5A5A	HYDROLASE	Endo-α-N-acetylgalactosaminidase ENDO-	3
2OSX	HYDROLASE	Endoglycoceramidase II	3
4AF9	CELL ADHESION	EPA1P	3
4ASL	CELL ADHESION	EPA1P	2
4D3W	CELL ADHESION	Epithelial adhesin 1	3
4W8B	HYDROLASE	Exo-xyloglucanase	8
5JOP	IMMUNE SYSTEM	Fab 14.22 light chain	4
2WMK	HYDROLASE	Fucolectin-related protein	5
2Z8F	SUGAR BINDING PROTEIN	Galacto/lacto-N-biose I transporter substrate-binding protein	4
3AP9	SUGAR BINDING PROTEIN	Galectin-8	5
5T7I	SUGAR BINDING PROTEIN	Galectin-8	4
4AW7	HYDROLASE	GH86A β -porphyranase	6
1WPC	HYDROLASE	Glucan 1,4-α-maltohexaosidase	4
4D6H	HYDROLASE	Glycoside hydrolase	4
4D6I	HYDROLASE	Glycoside hydrolase	4
4D6J	HYDROLASE	Glycoside hydrolase	4
4D71	HYDROLASE	Glycoside hydrolase	4
6ORH	HYDROLASE	Glycoside hydrolase	4
7UEN	IMMUNE SYSTEM	M86 antibody Fab light chain	2
1O7O	TRANSFERASE	N-acetyllactosaminide α-1,3-galactosyltransferase	2
5NRE	TRANSFERASE	N-acetyllactosaminide α-1,3-galactosyltransferase	2
4YFZ	VIRAL PROTEIN	Outer capsid protein VP4	4
5JSD	VIRAL PROTEIN	phiAB6 tailspike	8
3ILF	HYDROLASE	porphyranase A	4
2WT1	VIRAL PROTEIN	Putative fiber protein	4
5E1Q	HYDROLASE	Retaining α-galactosidase	3
6LF2	SUGAR BINDING PROTEIN	SeviL	4
6ORF	HYDROLASE	SpGH29	3
2VJJ	VIRAL PROTEIN	Tailspike protein	6
1MS9	HYDROLASE	Trans-sialidase	2
6P2M	HYDROLASE	Type 3a cellulose-binding domain protein	7
4W88	HYDROLASE	Xyloglucan-specific endo-beta-1,4-glucanase	4
2JEQ	HYDROLASE	Xyloglucanase	7
6MGL	HYDROLASE	Xyloglucanase	8

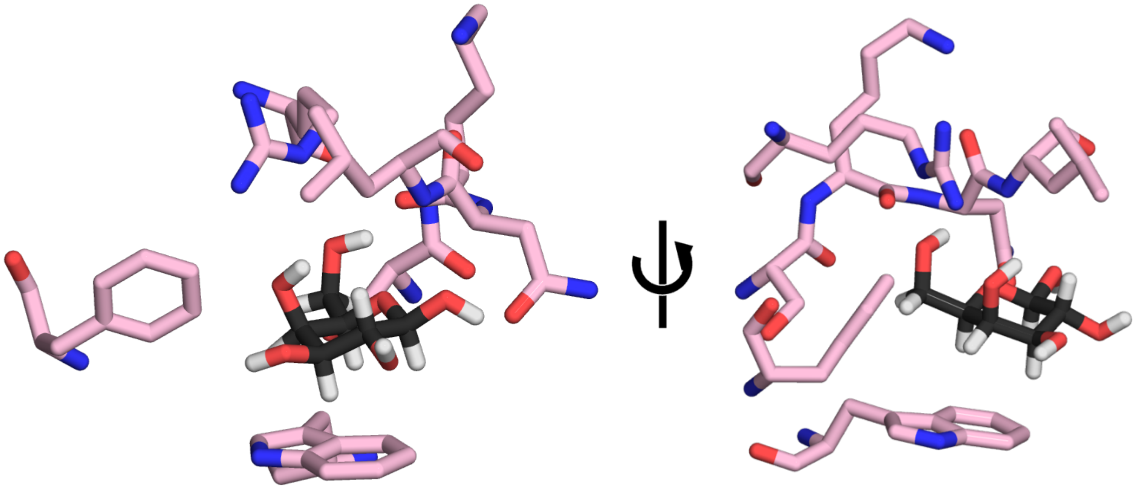

Figure S1. Visualization of the x-ray crystal structure of the progenitor toxin binding pocket with monomeric β-D-galactose bound (PDB ID 3AH4). The binding pocket is shown from two angles with an arrow depicting the direction of rotation. The carbohydrate atoms are colored as follows: carbon in black, oxygen in red, and hydrogen in white. Protein residues are shown with implicit hydrogen atoms and other atoms colored as follows: carbon in pink, oxygen in red, and nitrogen in blue.

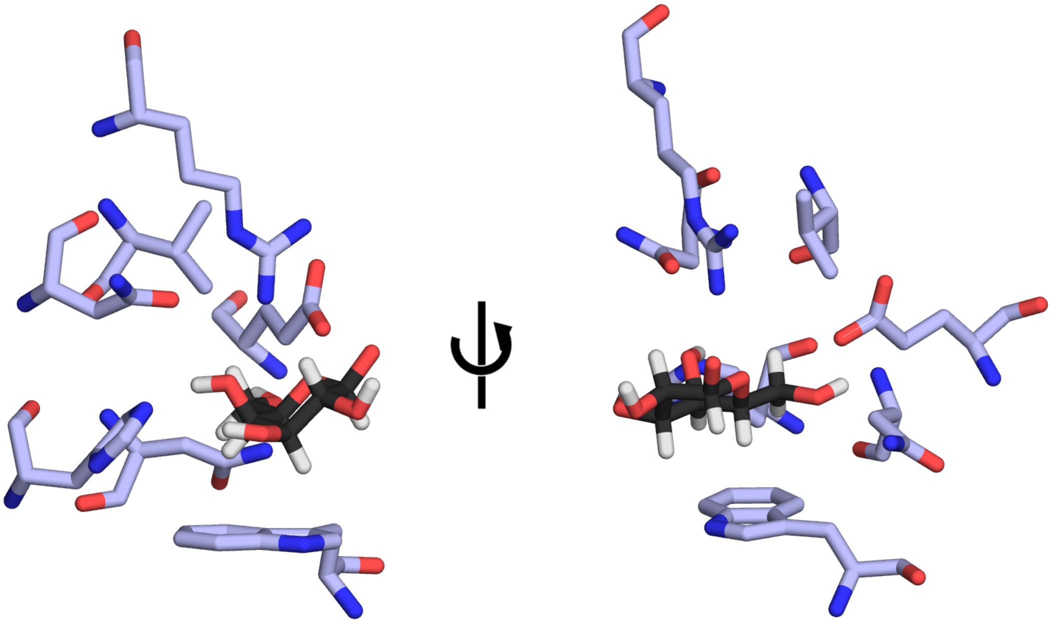

Figure S2. Visualization of the x-ray crystal structure of the galectin-3C binding pocket with monomeric β-D-galactose bound (PDB ID 3ZSJ). The binding pocket is shown from two angles with an arrow depicting the direction of rotation. The carbohydrate atoms are colored as follows: carbon in black, oxygen in red, and hydrogen in white. Protein residues are shown with implicit hydrogen atoms and other atoms colored as follows: carbon in pink, oxygen in red, and nitrogen in blue.

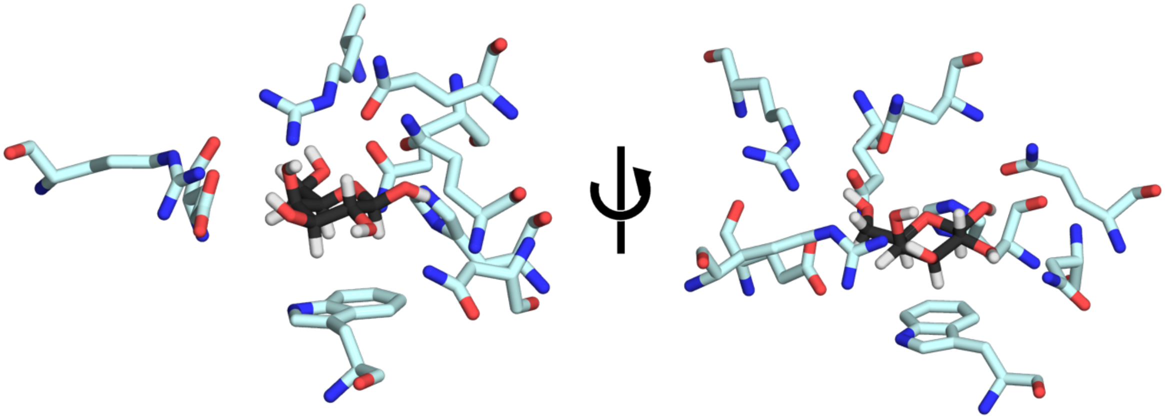

Figure S3. Visualization of the x-ray crystal structure of the galectin-10 binding pocket with monomeric β-D-galactose bound (PDB ID 6L67). The binding pocket is shown from two angles with an arrow depicting the direction of rotation. The carbohydrate atoms are colored as follows: carbon in black, oxygen in red, and hydrogen in white. Protein residues are shown with implicit hydrogen atoms and other atoms colored as follows: carbon in pink, oxygen in red, and nitrogen in blue.

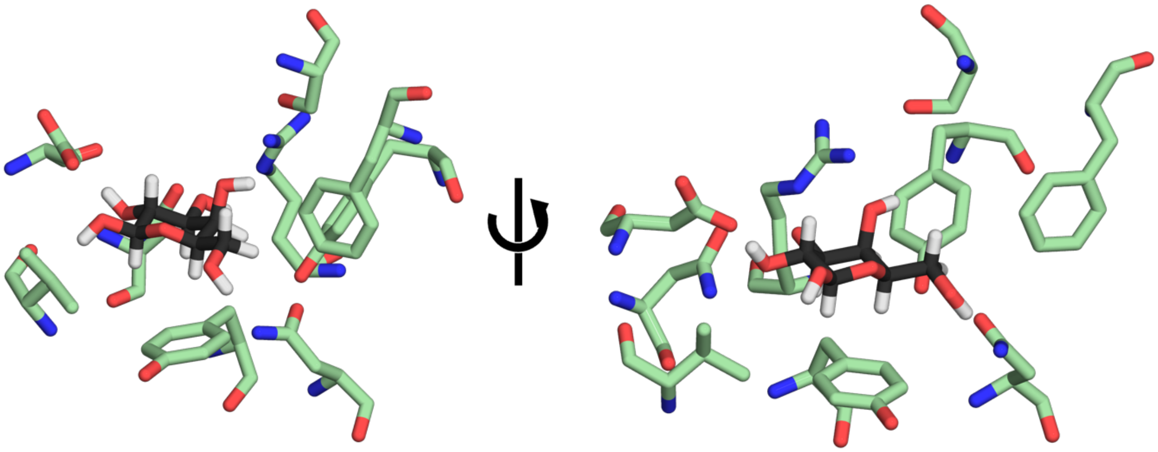

Figure S4. Visualization of the x-ray crystal structure of the pH6 antigen binding pocket with monomeric β-D-galactose bound (PDB ID 4F8L). The binding pocket is shown from two angles with an arrow depicting the direction of rotation. The carbohydrate atoms are colored as follows: carbon in black, oxygen in red, and hydrogen in white. Protein residues are shown with implicit hydrogen atoms and other atoms colored as follows: carbon in pink, oxygen in red, and nitrogen in blue.

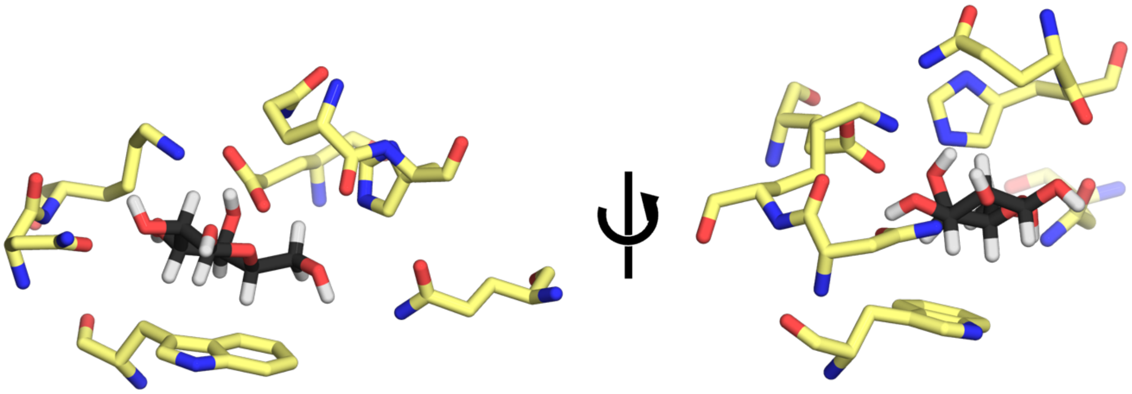

Figure S5. Visualization of the x-ray crystal structure of the cholera toxin binding pocket with monomeric β-D-galactose bound (PDB ID 2B3F). The binding pocket is shown from two angles with an arrow depicting the direction of rotation. The carbohydrate atoms are colored as follows: carbon in black, oxygen in red, and hydrogen in white. Protein residues are shown with implicit hydrogen atoms and other atoms colored as follows: carbon in pink, oxygen in red, and nitrogen in blue.

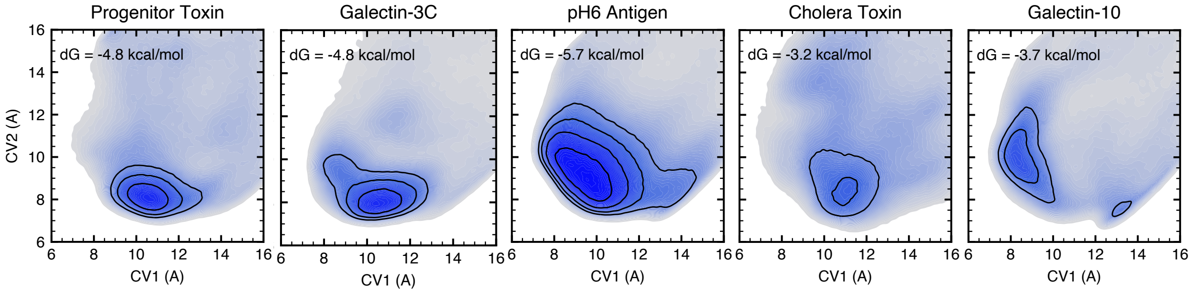

Figure S6. Free energy landscapes of β-D-galactose–protein binding interactions. All free energy landscapes were computed using MetaD, and the collective variables (CVs) are defined as follows: CV1 is $d_{\text{C1-Ctr}} + d_{\text{C2-Ctr}}$ and CV2 is $d_{\text{C4-Ctr}} + d_{\text{C6-Ctr}}$. Contour lines are drawn in black in 1 kcal/mol increments starting at -2 kcal/mol. All distances are reported in angstroms. The binding free energy (dG) is reported in kcal/mol.

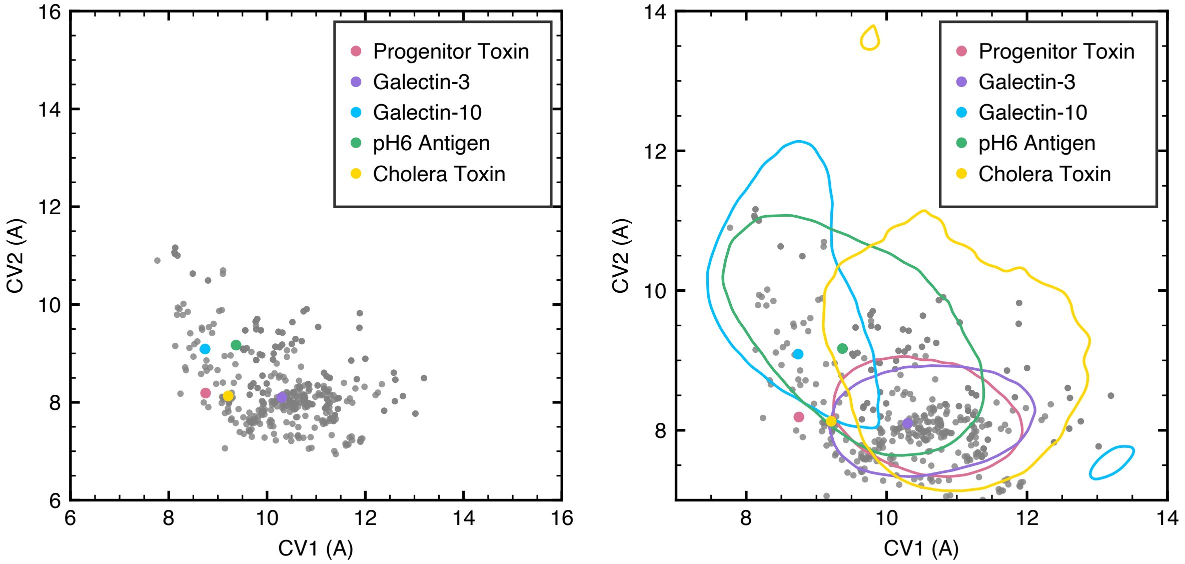

Figure S7. (left) Scatter plot of all β-D-galactose CH–π stacking interactions in protein-carbohydrate interactions crystalized in the Protein Data Bank. All interactions are colored in gray and plotted according to the two collective variables (CVs) defined as follows: CV1 is $d_{C1\text{-}Ctr} + d_{C2\text{-}Ctr}$ and CV2 is $d_{C4\text{-}Ctr} + d_{C6\text{-}Ctr}$. The CH–π stacking interactions analyzed in this study are colored as labeled in the legend. (right) The scatter plot from the left is overlaid with the contour lines of the corresponding metadynamics free energy landscapes. For each complex, the contiguous region within 1.5 kcal/mol of the most favorable orientation is encompassed by a contour line. All lines are colored as defined in the inset key.

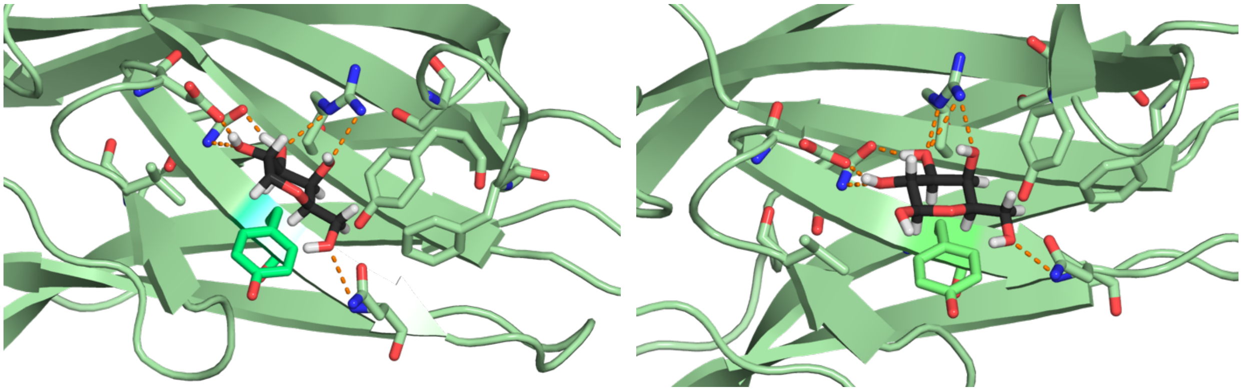

Figure S8. Visualization of pH6 antigen binding interactions to monomeric galactose. Two orientations that form distinct CH–π stacking interaction orientations are shown: (left) binding interaction with CH–π interactions formed by C–H groups 3, 4, 5, and 6 (right) and alternate binding position with CH–π interactions formed by C–H groups 1, 3, and 5. The carbohydrate atoms are colored as follows: carbon in black, oxygen in red, and hydrogen in white. Protein residues are shown with implicit hydrogen atoms and other atoms colored as follows: carbon in green, oxygen in red, and nitrogen in blue. Hydrogen bonds are represented as dotted orange lines.

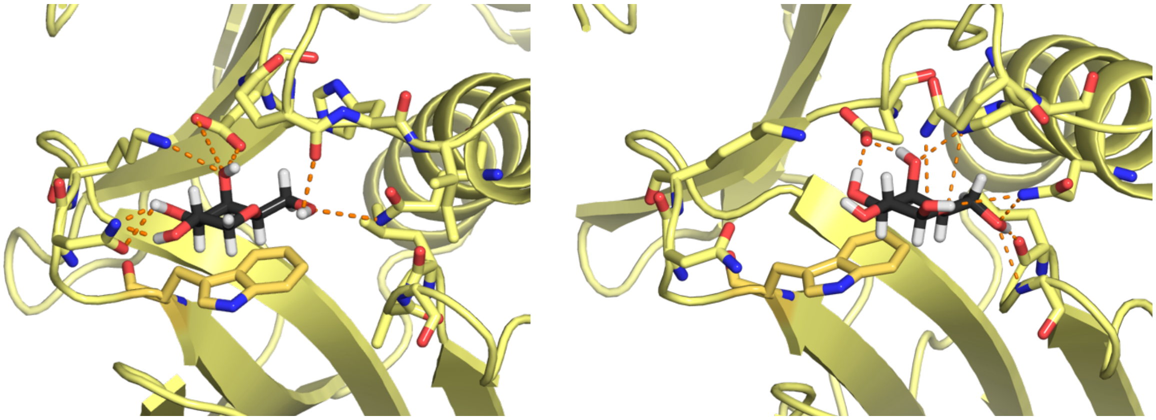

Figure S9. Visualization of cholera toxin binding interactions to monomeric galactose in two interaction positions. (Left) Binding interaction present in the crystal structure and (right) and alternate binding position observed in simulations. The carbohydrate atoms are colored as follows: carbon in black, oxygen in red, and hydrogen in white. Protein residues are shown with implicit hydrogen atoms and other atoms colored as follows: carbon in yellow, oxygen in red, and nitrogen in blue. Hydrogen bonds are represented as dotted orange lines.

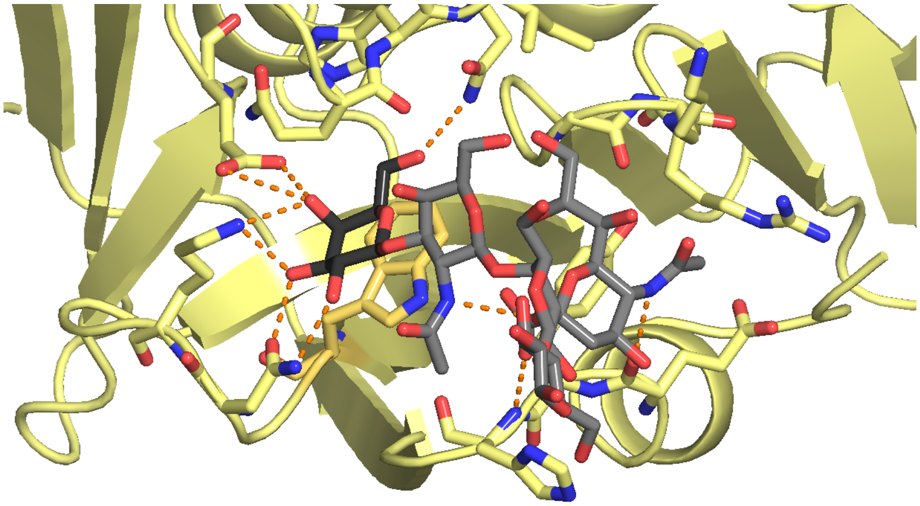

Figure S10. Visualization of the x-ray crystal structure of the cholera toxin binding interactions to GM1 pentasaccharide (PDB ID 2B3F). The carbohydrate atoms are colored as follows: terminal galactose carbon in black, all other carbon in gray, oxygen in red, and hydrogen in white. Protein residues are shown with implicit hydrogen atoms and other atoms colored as follows: carbon in yellow, oxygen in red, and nitrogen in blue. Hydrogen bonds are represented as dotted orange lines.

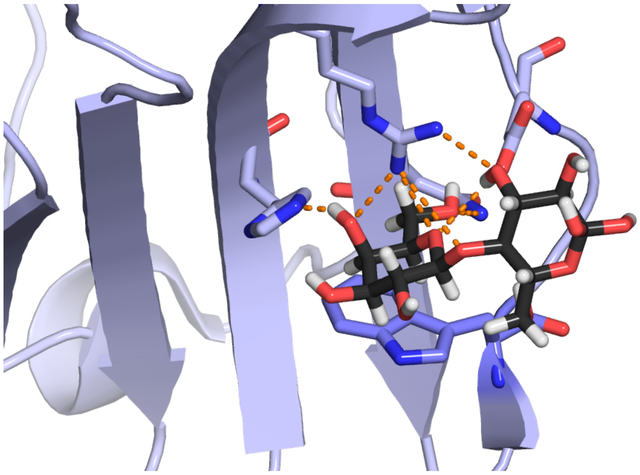

Figure S11. Visualization of the x-ray crystal structure of the galectin-3C binding interactions to lactose (PDB ID 3ZSJ). The carbohydrate atoms are colored as follows: carbon in black, oxygen in red, and hydrogen in white. Protein residues are shown with implicit hydrogen atoms and other atoms colored as follows: carbon in light blue, oxygen in red, and nitrogen in dark blue. Hydrogen bonds are represented as dotted orange lines.

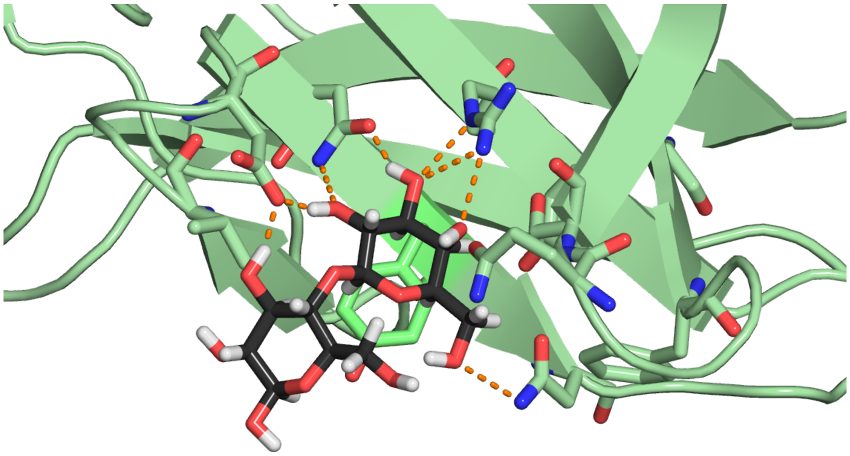

Figure S12. Visualization of the x-ray crystal structure of the pH6 antigen binding interactions to lactose (PDB ID 4F8O). The carbohydrate atoms are colored as follows: carbon in black, oxygen in red, and hydrogen in white. Protein residues are shown with implicit hydrogen atoms and other atoms colored as follows: carbon in green, oxygen in red, and nitrogen in blue. Hydrogen bonds are represented as dotted orange lines.

Table S2. MM/GBSA energies of the five monosaccharide binding interactions computed from unbiased molecular dynamics simulations reported along the MetaD minimum free energy of binding. All energies are reported in kcal/mol.

		MM/GBSA Energies					MetaD
Protein	PDB ID	van der Waals	Elst.	Polar Solv.	Non-polar Solv.	Total Energy	Min. Energy
Galectin-3C	3ZSJ	-13.7	-33.1	35.3	-2.5	-14.0	-4.7
Galectin-10	6L67	-16.4	-46.6	49.4	-3.0	-16.6	-3.7
Cholera	2CHB	-14.0	-39.0	45.8	-2.8	-10.0	-2.8
Botulinum	3AH4	-18.4	-38.5	38.8	-2.8	-20.8	-4.8
pH6 Antigen	4F8L	-14.4	-49.3	46.9	-3.1	-19.9	-3.7

Table S3. MM/GBSA energies of all oligosaccharide binding interactions computed from unbiased molecular dynamics simulations reported along the MetaD minimum free energy of binding. All energies are reported in kcal/mol.

		MM/GBSA Energies					MetaD
Protein	PDB ID	van der Waals	Elst.	Polar Solv.	Non-polar Solv.	Total Energy	Min. Energy
Galectin-3C	3ZSJ	-16.3	-70.9	64.3	-3.4	-26.4	-6.4
Cholera	2CHB	-43.2	-169.5	188.2	-6.7	-31.2	-7.6
pH6 Antigen	4F8L	-18.4	-60.6	61.4	-3.7	-21.3	-5.7

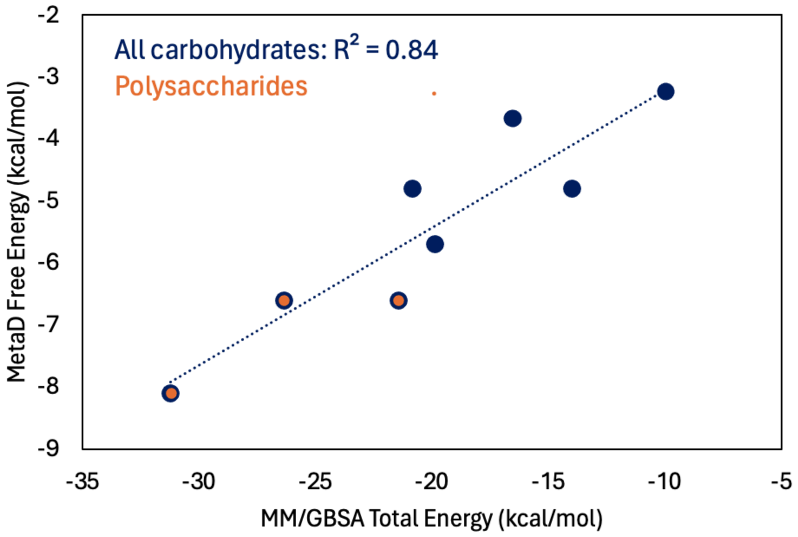

Figure S13. Parity plot of protein-carbohydrate binding interactions comparing the MM/GBSA total energy to the MetaD free energy, all reported in kcal/mol. All protein–carbohydrate complexes are included and colored in blue. Oligosaccharide complexes have an orange dot overlaid. A fit line for the full dataset is shown and the corresponding R^2 is reported.

Table S4. MM/GBSA energies of galectin-3C bound to galactose and lactose. All energies are decomposed into van der Waals (vdW), electrostatics (Elst), and solvation (Solv.: Gibbs Born (GB) + Solvent Accessible Surface Area (SASA)) energies for each residue. The difference in total energy contribution from each residue is reported as lactose total energy – galactose total energy. All energies are reported in kcal/mol.

Galactose (Monosaccharide) binding					Lactose (Disaccharide) binding					Difference
	vdW	Elst	Solv	Total		vdW	Elst	Solv	Total	Total
ARG 144	-0.2	-1.2	1.0	-0.4	ARG 144	-0.2	-1.6	1.4	-0.5	-0.1
HIS 158	-0.5	-3.9	2.7	-1.7	HIS 158	-0.5	-3.8	2.6	-1.8	-0.1
ASN 160	-0.6	0.8	-0.1	0.1	ASN 160	-0.6	1.3	-0.6	0.2	0.1
ARG 162	-1.0	-3.7	2.2	-2.5	ARG 162	-0.6	-13.2	7.2	-6.6	-4.1
GLU 165	-0.1	0.5	-0.4	0.0	GLU 165	-0.3	3.6	-3.0	0.2	0.2
VAL 172	-0.5	-0.1	0.1	-0.6	VAL 172	-0.8	-0.2	0.0	-1.0	-0.4
ASN 174	-0.2	-3.3	0.7	-2.8	ASN 174	-0.3	-3.2	0.2	-3.2	-0.4
TRP 181	-2.6	-0.9	0.5	-3.0	TRP 181	-2.9	-0.7	0.4	-3.2	-0.2
GLU 184	-0.1	-4.9	5.6	0.5	GLU 184	0.1	-15.2	15.6	0.5	0
ARG 186	-0.1	0.2	-0.2	-0.1	ARG 186	-0.6	-2.2	1.2	-1.6	-1.5

Table S5. MM/GBSA energies of cholera toxin bound to galactose and GM1 pentasaccharide. All energies are decomposed into van der Waals (vdW), electrostatics (Elst), and solvation (Solv.: Gibbs Born (GB) + Solvent Accessible Surface Area (SASA)) energies for each residue. The difference in total energy contribution from each residue is reported as lactose total energy – galactose total energy. All energies are reported in kcal/mol.

Galactose (Monosaccharide) binding					GM1 Pentasaccharide binding					Difference
	vdW	Elst	Solv	Total		vdW	Elst	Solv	Total	Total
TYR 12	-0.2	-0.1	0.3	-0.1	TYR 115	-4.1	-6.6	5.0	-5.7	-5.6
HIS 13	-0.2	-0.3	0.4	-0.1	HIS 116	-2.4	-4.4	3.2	-3.5	-3.4
ASN 14	-0.1	-0.2	0.2	-0.1	ASN 117	-0.6	-1.0	1.3	-0.3	-0.2
GLU 51	0.5	-11.8	9.7	-1.7	GLU 154	0.5	0.8	-2.7	-1.4	0.3
GLN 56	-0.4	-0.7	0.5	-0.6	GLN 159	-1.3	-1.6	1.7	-1.2	-0.6
HIS 57	-0.8	-0.6	0.3	-1.1	HIS 160	-1.0	-1.0	0.8	-1.1	0
GLN 61	-0.4	-1.0	0.6	-0.8	GLN 164	-0.4	-1.6	1.1	-0.9	-0.1
TRP 88	-2.2	-0.8	0.6	-2.4	TRP 191	-3.2	-3.5	2.7	-4.1	-1.7
ASN 90	-0.2	-1.4	0.9	-0.7	ASN 193	-0.3	-4.1	2.7	-1.8	-1.1
LYS 91	-0.4	-1.1	0.8	-0.7	LYS 194	-0.5	-15.2	14.5	-1.2	-0.5
GLY 136	-0.2	-0.2	0.2	-0.3	GLY 239	-1.4	-0.7	0.9	-1.2	-0.9
LYS 137	0.0	-0.1	0.2	0.0	LYS 240	-2.2	-22.3	22.8	-1.6	-1.6
ARG 138	0.0	0.0	0.0	0.0	ARG 241	-0.9	-12.7	13.4	-0.3	-0.3

Table S6. MM/GBSA energies of pH6 antigen bound to galactose and lactose. All energies are decomposed into van der Waals (vdW), electrostatics (Elst), and solvation (Solv.: Gibbs Born (GB) + Solvent Accessible Surface Area (SASA)) energies for each residue. The difference in total energy contribution from each residue is reported as lactose total energy – galactose total energy. All energies are reported in kcal/mol.

Galactose (Monosaccharide) binding					Lactose (Disaccharide) binding					Difference
	vdW	Elst	Solv	Total		vdW	Elst	Solv	Total	Total
ARG 40	-0.2	-10.3	5.8	-4.7	ARG 41	-0.3	-9.5	5.3	-4.5	0.2
VAL 70	-0.9	0.1	-0.2	-1	VAL 71	-1.3	0.0	-0.2	-1.5	-0.5
ASP 73	0.1	-8.7	8.5	-0.1	ASP 74	0.3	-14.4	14.1	0.1	0.2
ASN 75	-0.1	-4.1	1.7	-2.5	ASN 76	-0.1	-4.1	1.8	-2.4	0.1
TYR 77	-2.5	-0.9	1	-2.4	TYR 78	-3.4	-1.2	1.7	-2.9	-0.5
ASN 79	-0.5	-2.0	1.1	-1.4	ASN 80	-0.5	-2.6	1.4	-1.8	-0.4
TYR 112	-1.3	0.1	0.3	-0.9	TYR 113	-1.4	0.1	0.2	-1	-0.1
PHE 114	-0.4	0.0	0	-0.4	PHE 115	-0.5	0.0	0.1	-0.5	-0.1
SER 119	-0.4	-0.6	0.9	-0.1	SER 120	-0.4	-0.4	0.7	0.0	0.1

Table S7. MM/GBSA energies of progenitor toxin bound to galactose. All energies are decomposed into van der Waals (vdW), electrostatics (Elst), and solvation (Solv.: Gibbs Born (GB) + Solvent Accessible Surface Area (SASA)) energies for each residue. All energies are reported in kcal/mol.

	vdW	Elst	Solv	Total
SER 164	-0.4	0.1	-0.1	-0.5
LYS 165	0.1	-4.1	2.5	-1.5
ASN 166	-1	-4.9	2.9	-3
LEU 167	-2	-1.2	0.7	-2.5
TRP 175	-2.8	-0.9	0.2	-3.5
PHE 178	-0.9	-0.5	0.5	-0.9
ARG 182	-0.6	-9.5	5.8	-4.3

Table S8. MM/GBSA energies of galectin-10 bound to galactose. All energies are decomposed into van der Waals (vdW), electrostatics (Elst), and solvation (Solv.: Gibbs Born (GB) + Solvent Accessible Surface Area (SASA)) energies for each residue. All energies are reported in kcal/mol.

	vdW	Elst	Solv	Total
GLN 35	-0.3	0.1	-0.2	-0.4
HIS(+) 51	-0.8	-7.7	5.6	-3.0
GLN 53	-0.7	0.5	0.2	-0.1
ASN 63	-0.4	-3.2	1.1	-2.6
TRP 70	-2.9	-0.4	-0.2	-3.5
GLN 73	-0.5	-2.9	2.3	-1.1
ARG 126	-0.3	-0.8	-0.1	-1.2
GLU 169	-0.5	-11.9	11.3	-1.1
ARG 196	-0.4	1.9	-2.1	-0.5

Text S1. QM Cluster model preparation. To investigate the energetics of the galactotriose translocation from orientation A to orientation B in TmCBM61, we constructed QM cluster models using the coordinates from PDB entries 2XON and 2XOM. These PDB structures were selected because 2XON captures the galactotriose in orientation A and 2XOM captures the galactotriose in orientation B. Two QM cluster models were constructed containing residues 1) GAL1, GAL2, GAL3, N56, N66, W68, W123, W154, F156 for a cluster model capturing all protein–ligand contacts (198 atoms) and 2) GAL1, GAL2, GAL3, W68, W123, and W154 capturing only the CH-π interactions between the Galactotriose and tryptophan (144 atoms). All amino acid coordinates were taken from 2XON. To obtain the coordinates of the Galactotriose and the two orientations, 2XOM and 2XON were aligned using the residues N56, N66, W68, W123, W154, F156 as a mask using ChimeraX. From the aligned structures, the coordinates of the galactotriose in 2XOM were used for orientation A and the coordinates from 2XON were used for orientation B. The backbone atoms for each residue were included and capped with hydrogen atoms.

Text S2. QM cluster model calculations. QM calculations for galactotriose translocation energetics were performed using the GPU-accelerated quantum chemistry software TeraChem (developer version 1.9).[1] Density functional theory (DFT) calculations were carried out at the B3LYP[2,3]/6-31G* level with Grimme's D3[4] (BJ[5]) dispersion corrections to account for long-range interactions. Implicit solvent effects were modeled using the conductor-like polarizable continuum model (C-PCM)[6,7] with a dielectric constant of 10 to approximate the electrostatic environment of the carbohydrate binding site. Following geometry optimization of intermediates, reaction pathways were explored by interpolating 24 images between adjacent states using the image-dependent pair potential (IDPP) method[8] in ORCA[9,10] version 6.0.0. Only the interpolated structures were obtained from ORCA in order to obtain a good initial guess. These interpolated images then served as an initial guess for the minimum energy path, which was then optimized using the climbing image nudged elastic band (CI-NEB)[11] method in TeraChem. The NEB calculations included 24 images, with fixed endpoints at their optimized geometries and variable spring constants ranging from 0.01 to 0.1 atomic units. To preserve the overall structure of the binding site, heavy atoms in the protein backbone were constrained to their crystallographic positions during CI-NEB optimization. The transition state energy estimates were derived from the highest-energy climbing image. Given the size of the clusters, thermodynamic corrections were not applied, and comparisons were based on electronic energies.

Table S9. Description of the QM cluster models used to calculate the galactotriose translocation energetics with and without non-tryptophan amino acids. The QM cluster model name, the total atom count, the net charge of the cluster, and the residues included in each model are listed. All amino acid positions and the reactant state (orientation A) of the galactotriose were constructed from PDB:2XON, while the product conformation (orientation B) of the galactotriose was constructed using PDB:2XOM.

**QM cluster model**	**Atom count**	**Charge**	**Residues**
All-residue model	198	0	GAL1, GAL2, GAL3, N56, N66, W68, W123, W154, F156
Trp-only model	144	0	GAL1, GAL2, GAL3, W68, W123, W154

Table S10. Description of the three steps as assessed via CI-NEB for the transition of a galactotriose from orientation A to orientation B. The intermediates and putative transition states of the step are listed alongside a description of the key atom movements that occur.

**Step**	**Translocation step description**
**$R \rightarrow TS_1 \rightarrow IM_1$**	Rotation of GAL2 OH3
**$IM_1 \rightarrow TS_2 \rightarrow IM_2$**	Rotation of GAL2 C6-OH6
**$IM_1 \rightarrow TS_3 \rightarrow P$**	Translocation of GAL trimer with N56 and N66 hydrogen bond breaking and reformation

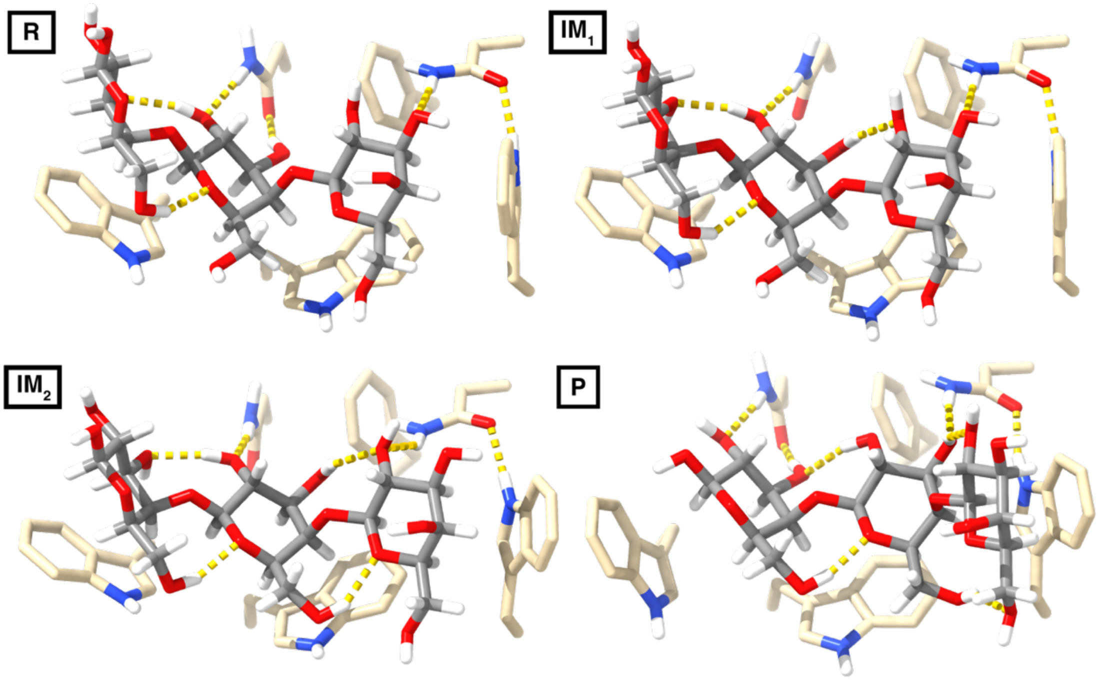

Figure S14. Structures of intermediates in translocation of the GAL trimer from orientation A to orientation B. (top left) Structure of the reactant state (**R**). (top right) Structure of intermediate 1 (**IM$_1$**). (bottom left) Structure of intermediate 2 (**IM$_2$**). (bottom right) Structure of the product state (**P**). Atoms are colored as follows: GAL trimer carbon in gray, protein carbons in wheat, nitrogen in blue, oxygen in red, hydrogen in and white.

Table S11. Tabulated reaction energetics of all intermediates and putative transition states for the transition of galactotriose from orientation A to orientation B. Geometries were optimized at the B3LYP-D3(BJ)/6-31G* level of theory and putative transition states were identified using CI-NEB calculations in TeraChem. Relative energies are reported in kcal/mol.

**Step**	**All-residue QM cluster model ΔE (kcal/mol)**	**Trp-only QM cluster model ΔE (kcal/mol)**
**R**	0.00	0.00
**TS$_1$**	6.58	1.47
**IM$_1$**	0.44	-3.98
**TS$_2$**	8.09	3.70
**IM$_2$**	-2.81	-7.44
**TS$_3$**	8.16	0.45
**P**	-8.96	-10.31

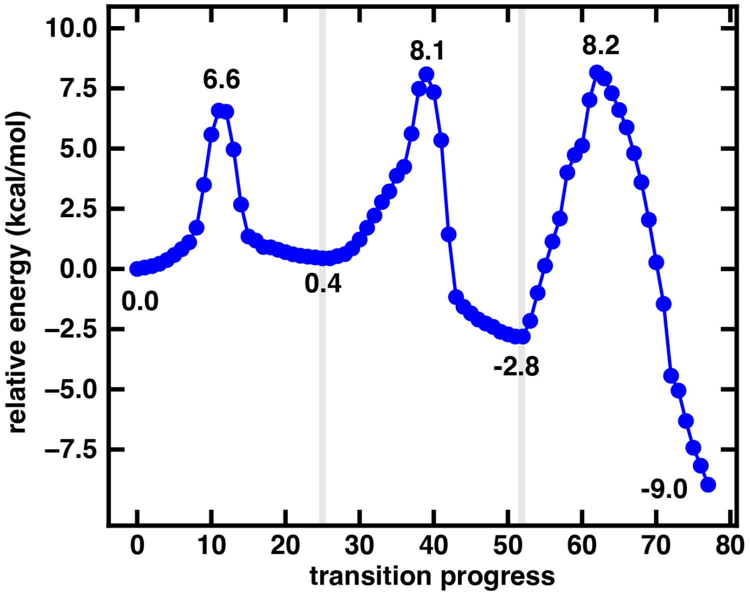

Figure S15. Potential energy surface from CI-NEB calculations for the galactotriose translocation from orientation A to orientation B with the all-residue QM cluster model. The relative energies are shown in kcal/mol. The relative energy of the highest-energy point is labeled. The x-axis indicates the translocation progress from orientation A to orientation B through the number of NEB steps. The divisions between separate NEB calculations are shown with vertical gray lines.

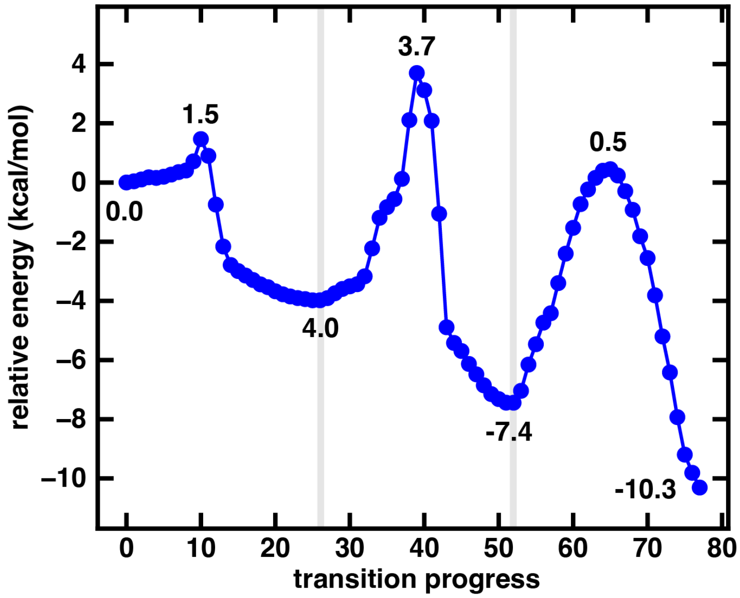

Figure S16. Potential energy surface from NEB calculations for the galactotriose translocation from orientation A to orientation B with the Trp-only QM cluster model. The relative energies are shown in kcal/mol. The relative energy of the highest-energy point is labeled. The x-axis indicates the reaction progress through the number of NEB steps. The divisions between separate NEB calculations are shown with vertical gray lines.

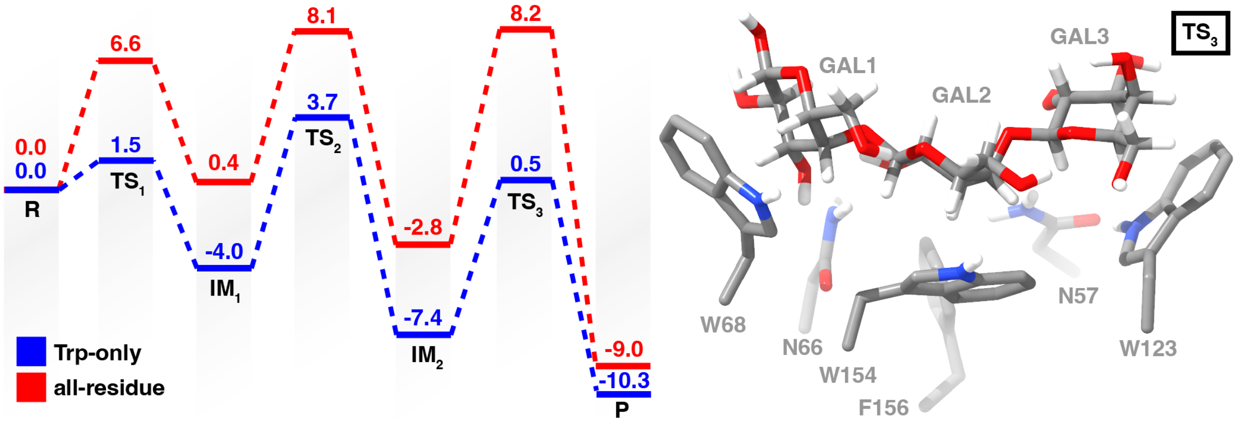

Figure S17. (left) Reaction energy diagram for transition of the Galactotriose from orientation A to orientation B calculated with the all-residue QM cluster model (red) and the Trp-only QM cluster model (blue). Energies are shown in kcal/mol with the intermediates and transition states labeled. (right) Visualization of the highest-energy putative transition state with the residues labeled in gray. Atoms are colored as follows: carbon in gray, nitrogen in blue, oxygen in red, hydrogen in and white.

Table S12. Protein and carbohydrate ligand names for each system analyzed. The protein charge and number of atoms are reported for each system. Simulation details are also reported for all mutant proteins with specified mutations.

PDB ID	Protein	Mutation	Ligand	Protein Charge (e)	System Size (atoms)
3ZSJ	Galectin-3	N/A	Lactose	+5	23302
3ZSJ	Galectin-3	His158Ala	Lactose	+5	23295
3ZSJ	Galectin-3	Glu184Ala	Lactose	+6	23295
3ZSJ	Galectin-3	N/A	β-D-Galactose	+5	23308
4F8O	pH6 Antigen	N/A	Lactose	-3	25284
4F8L	pH6 Antigen	N/A	β-D-Galactose	-3	25631
2CHB	Cholera toxin	N/A	β-D-Galactose	+5	55872
2CHB	Cholera toxin	N/A	GM1 pentasaccharide	+4	61445
3AH4	Botulinum toxin	N/A	β-D-Galactose	+3	39551
2XON	TmCBM61	N/A	β-1,4-galactotriose	+0	21384
6L67	Galectin-10	N/A	β-D-Galactose	+0	31757

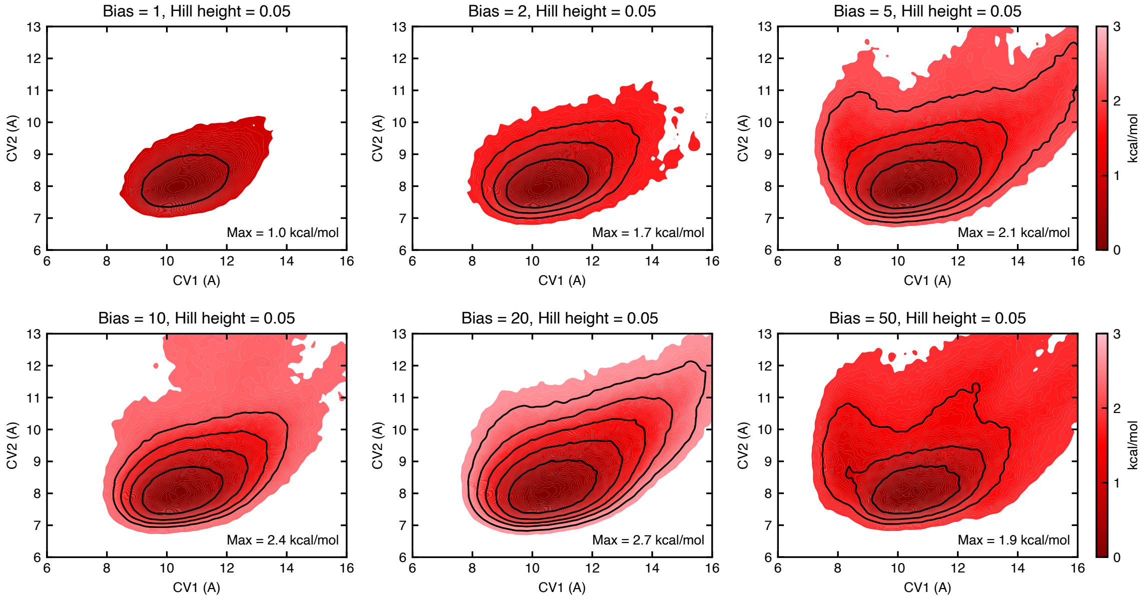

Figure S18. Metadynamics free energy landscapes of galectin-3C computed with varying bias factors and a hill height of 0.05 as reported at the top of each plot. Each plot represents a single 150 ns simulation. Collective variables (CVs) are defined as follows: CV1 is $d_{\text{C1-Ctr}} + d_{\text{C2-Ctr}}$ and CV2 is $d_{\text{C4-Ctr}} + d_{\text{C6-Ctr}}$. Contour lines are drawn in black in 0.5 kcal/mol increments starting at 0.5 kcal/mol. All distances are reported in angstroms. The maximum energy difference between sampled orientations is reported in kcal/mol.

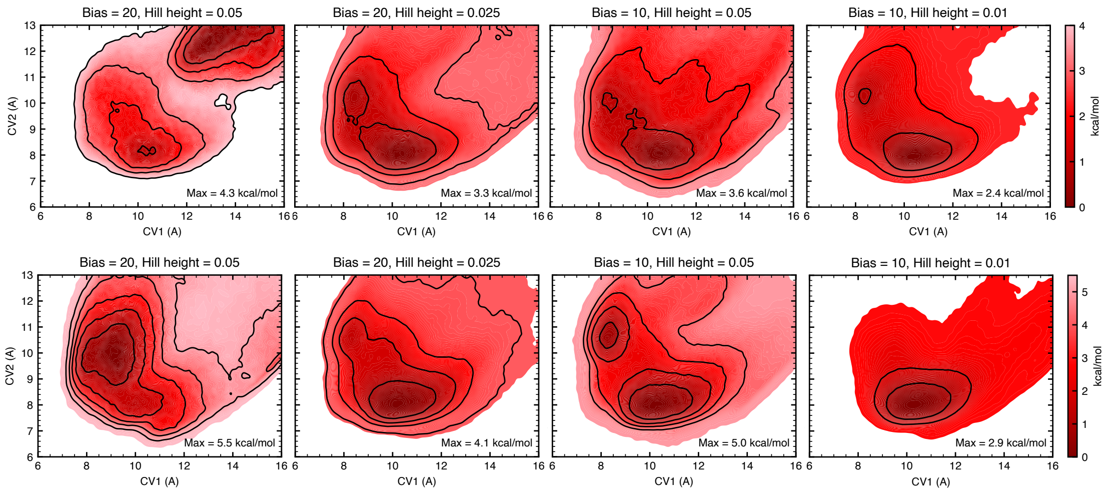

Figure S19. Metadynamics free energy landscapes of galectin-3C mutants (top) His158Ala (158A) and (bottom) Glu184Ala (E184A) computed with varying bias and hill heights reported at the top of each plot. Collective variables (CVs) are defined as follows: CV1 is $d_{\text{C1-Ctr}} + d_{\text{C2-Ctr}}$ and CV2 is $d_{\text{C4-Ctr}} + d_{\text{C6-Ctr}}$. Contour lines are drawn in black in 0.5 kcal/mol increments starting at 0.5 kcal/mol. All distances are reported in angstroms. The maximum energy difference between sampled orientations is reported in kcal/mol.

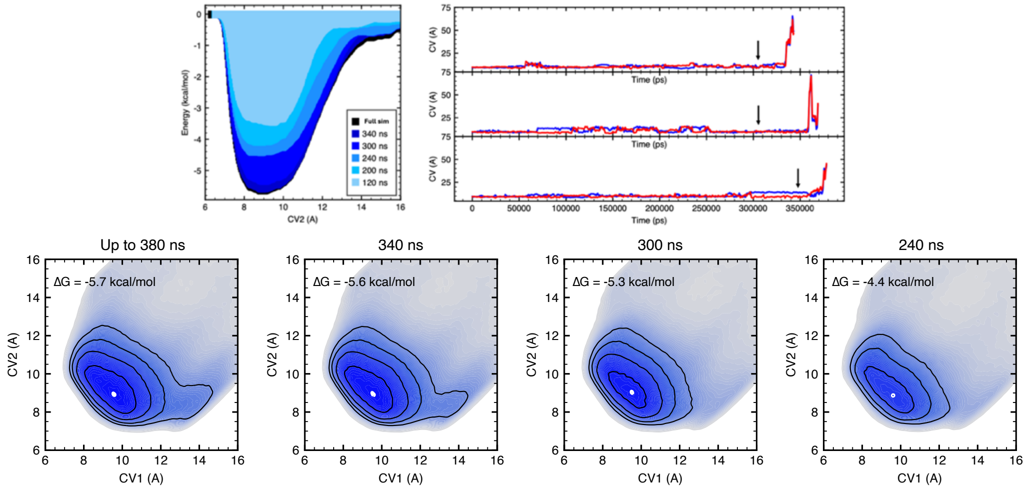

Figure S20. Analysis of the convergence of the WT-MetaD simulation of the pH6 antigen-β-D-galactose complex over time. Collective variables (CVs) are defined as follows: CV1 is $d_{\text{C1-Ctr}} + d_{\text{C2-Ctr}}$, and CV2 is s $d_{\text{C4-Ctr}} + d_{\text{C6-Ctr}}$, where ‘C1’, ‘C2’, ‘C4’, and ‘C6’ are the corresponding carbon atoms on galactose and ‘Ctr’ is the nearest amino acid centroid. (top left) Convergence of collective variable 2 (CV2) with simulation progress. The filled energy wells as of the first 120, 200, 240, 300, 340, and all (340-380) ns are plotted. (top right) Time-averaged trace of CV1 (blue) and CV2 (red) over each of the three independent metadynamics (MetaD) simulations. Black arrows represent the release of upper wall constraints on CV1 and CV2 to allow ligand dissociation. (bottom) Resulting energy landscapes from the full simulations and the first 340, 300, and 240 ns of the MetaD simulations, respectively. Contour lines that encompass the minimum energy orientation are drawn in black in 1 kcal/mol increments starting at -2 kcal/mol, and the white dot represents the minimum energy orientation. All energies are reported in kcal/mol.

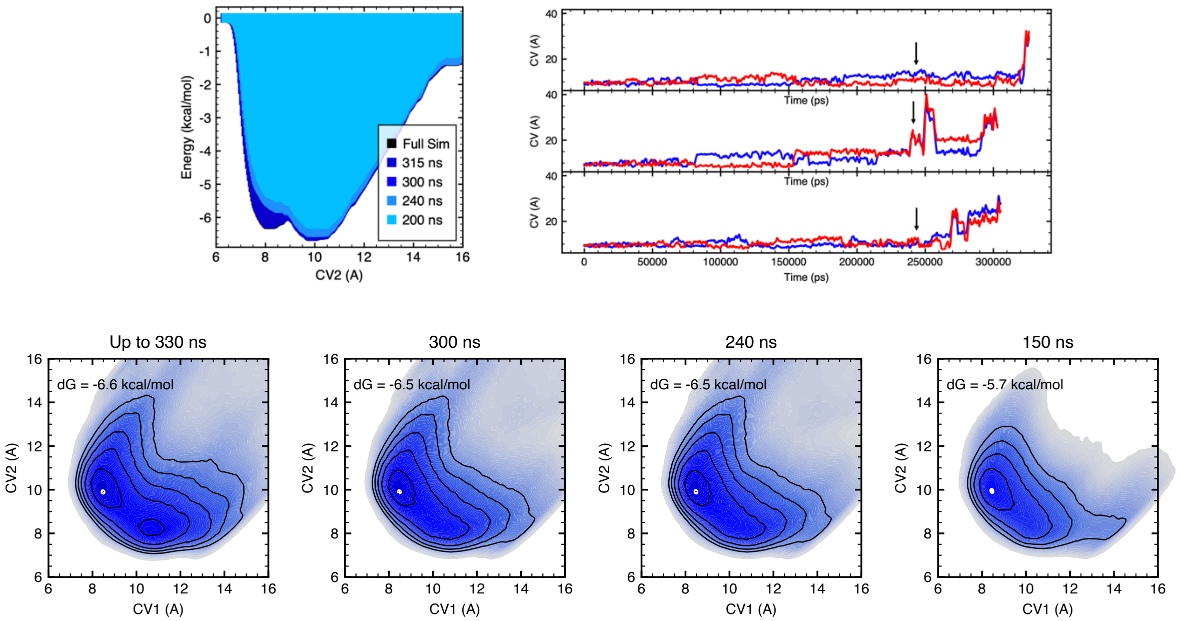

Figure S21. Analysis of the convergence of the WT-MetaD simulation of the pH6 antigen-lactose complex over time. Collective variables (CVs) are defined as follows: CV1 is $d_{\text{C1-Ctr}} + d_{\text{C2-Ctr}}$, and CV2 is s $d_{\text{C4-Ctr}} + d_{\text{C6-Ctr}}$, where 'C1', 'C2', 'C4', and 'C6' are the corresponding carbon atoms on galactose and 'Ctr' is the nearest amino acid centroid. (top left) Convergence of collective variable 2 (CV2) with simulation progress. The filled energy wells as of the first 200, 240, 300, 315, and all (308-328) ns are plotted. (top right) Time-averaged trace of CV1 (blue) and CV2 (red) over each of the three independent metadynamics (MetaD) simulations. Black arrows represent the release of upper wall constraints on CV1 and CV2 to allow ligand dissociation. (bottom) Resulting energy landscapes from the full simulations and the first 315, 300, and 240 ns of the MetaD simulations, respectively. Contour lines that encompass the minimum energy orientation are drawn in black in 1 kcal/mol increments starting at -2 kcal/mol, and the white dot represents the minimum energy orientation. All energies are reported in kcal/mol.

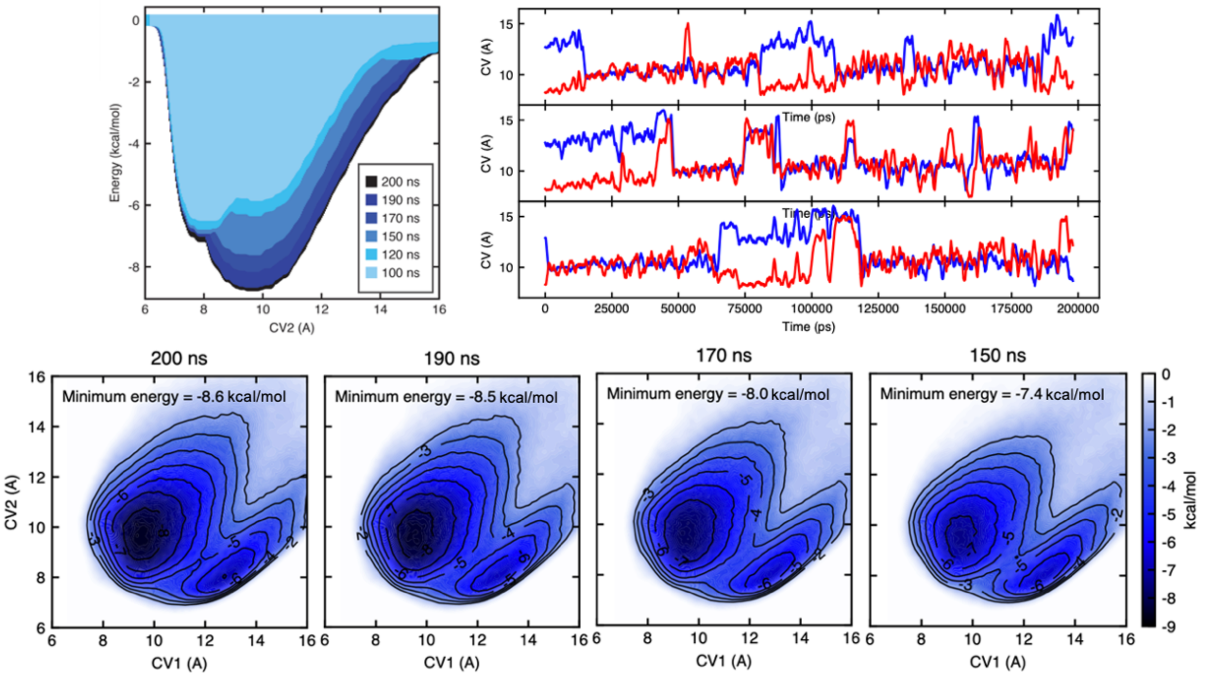

Figure S22. Analysis of the convergence of a MetaD free energy landscape of a *Thermotoga maritima* processive carbohydrate binding module (TmCBM61) over time. (top left) Convergence of collective variable 2 (CV2) with simulation progress. The filled energy wells as of the first 100, 120, 150, 170, 190, 200 ns are plotted. (top right) Time averaged trace of CV1 in blue and CV2 in red over each of the three independent MetaD simulations. CV1 is defined as $d_{C1\text{-}Ctr} + d_{C2\text{-}Ctr}$, and CV2 is defined as $d_{C4\text{-}Ctr} + d_{C6\text{-}Ctr}$, where 'C1', 'C2', 'C4', and 'C6' are the corresponding carbon atoms on galactose and 'Ctr' is the nearest amino acid centroid. (bottom) Resulting energy landscapes from the first 200, 190, 170, 150 ns of the three averaged MetaD simulations, respectively. Contour lines that encompass the minimum energy orientation are drawn in black in 1 kcal/mol increments starting at -2 kcal/mol, and the white dot represents the minimum energy orientation. All energies are reported in kcal/mol.

Table S13. Selection of amino acids included in MM/GBSA energy decomposition analysis of binding interactions.

PDB ID	Protein	Amino Acids
3ZSJ	Galectin-3	Arg 32, His 46, Asn 48, Arg 50, Glu 53, Val 60, Asn 62, Trp 69, Glu 72, Arg 74
4F8L	pH6 Antigen	Arg 40, Val 70, Asp 73, Asn 75, Tyr 77, Asn 79, Tyr 112, Phe 114, Ser 119
4F8O	pH6 Antigen	Arg 41, Val 71, Asp 74, Asn 76, Tyr 78, Asn 80, Tyr 113, Phe 115, Ser 120
2CHB Gal	Cholera toxin	Asn 14, Glu 51, Gln 56, His 57, Gln 61, Trp 88, Asn 90, Lys, 91
2CHB GM1	Cholera toxin	Tyr 115, His 116, Asn 117, Glu 154, Gln 159, His 160, Gln 164, Trp 191, Asn 193, Lys 194, Gly 239, Lys 240, Arg 241
3AH4	Progenitor toxin	Ser 164, Lys 165, Asn 166, Leu 167, Trp 175, Phe 178, Arg 182
6L67	Galectin-10	Gln 35, His 51, Gln 53, Asn 63, Trp 70, Gln 73, Arg 126, Glu 169, Arg 196

Table S14. Number of frames included in each MM/GBSA calculation.

PDB ID	Protein	Mutation	Ligand	# Frames
3ZSJ	Galectin-3	N/A	Lactose	572
3ZSJ	Galectin-3	N/A	β-D-Galactose	555
6L67	Galectin-10	N/A	β-D-Galactose	524
4F8O	pH6 Antigen	N/A	Lactose	567
4F8L	pH6 Antigen	N/A	β-D-Galactose	485
2CHB	Cholera toxin	N/A	β-D-Galactose	525
2CHB	Cholera toxin	N/A	GM1 pentasaccharide	744
3AH4	Botulinum toxin	N/A	β-D-Galactose	572

References

(1) Seritan, S.; Bannwarth, C.; Fales, B. S.; Hohenstein, E. G.; Isborn, C. M.; Kokkila-Schumacher, S. I. L.; Li, X.; Liu, F.; Luehr, N.; Snyder, J. W.; Song, C. C.; Titov, A. V.; Ufimtsev, I. S.; Wang, L. P.; Martinez, T. J. TeraChem: A graphical processing unit-acceleratedelectronic structure package forlarge-scaleab initio molecular dynamics. *Wiley Interdiscip. Rev. Comput. Mol. Sci.* **2021,** *11*.

(2) Lee, C.; Yang, W.; Parr, R. G. Development of the Colle-Salvetti correlation-energy formula into a functional of the electron density. *Phys Rev B Condens Matter* **1988,** *37*, 785-789.

(3) Becke, A. D. Density-Functional Thermochemistry .3. The Role of Exact Exchange. *J Chem Phys* **1993,** *98*, 5648-5652.

(4) Grimme, S.; Antony, J.; Ehrlich, S.; Krieg, H. A consistent and accurate ab initio parametrization of density functional dispersion correction (DFT-D) for the 94 elements H-Pu. *J Chem Phys* **2010,** *132*, 154104.

(5) Grimme, S.; Ehrlich, S.; Goerigk, L. Effect of the Damping Function in Dispersion Corrected Density Functional Theory. *J Comput Chem* **2011,** *32*, 1456-1465.

(6) Lange, A. W.; Herbert, J. M. A smooth, nonsingular, and faithful discretization scheme for polarizable continuum models: The switching/Gaussian approach. *The Journal of Chemical Physics* **2010,** *133*.

(7) Liu, F.; Luehr, N.; Kulik, H. J.; Martínez, T. J. Quantum Chemistry for Solvated Molecules on Graphical Processing Units Using Polarizable Continuum Models. *J Chem Theory Comput* **2015,** *11*, 3131-44.

(8) Smidstrup, S.; Pedersen, A.; Stokbro, K.; Jónsson, H. Improved initial guess for minimum energy path calculations. *The Journal of Chemical Physics* **2014,** *140*.

(9) Neese, F.; Wennmohs, F.; Becker, U.; Riplinger, C. The ORCA quantum chemistry program package. *J. Chem. Phys.* **2020,** *152*.

(10) Neese, F. The ORCA program system. *WIREs Computational Molecular Science* **2012,** *2*, 73-78.

(11) Henkelman, G.; Uberuaga, B. P.; Jónsson, H. A climbing image nudged elastic band method for finding saddle points and minimum energy paths. *The Journal of Chemical Physics* **2000,** *113*, 9901-9904.
