# Supplementary material for: CH−π interactions confer orientational flexibility in protein–carbohydrate binding sites
Source: J Biol Chem. 2025 Jun 14;301(8):110379. doi: 10.1016/j.jbc.2025.110379 (PMC12309604; doi:10.1016/j.jbc.2025.110379)
Supplement: Supporting information [file mmc2.docx]

Simulation input information; effect of metadynamics bias factor on energetic landscape, effect of metadynamics hill height on energetic landscapes; WT-MetaD convergence with simulation progress – monosaccharide; WT-MetaD convergence with simulation progress – oligosaccharide; WT-MetaD convergence with simulation progress – TmCBM61; amino acids involved in MM/GBSA decomposition; frames used for MM/GBSA calculations; proteins with multiple CH- stacking interactions; progenitor toxin binding pocket visualization; galectin-3C binding pocket visualization; galectin-10 binding pocket visualization; pH6 antigen binding pocket visualization; cholera toxin binding pocket visualization; monosaccharide free energy landscapes; monosaccharide free energy landscapes *versus* crystal structures; cholera toxin–galactose binding orientation comparison; pH6 antigen–galactose binding orientation comparison; cholera toxin–GM1 pentasaccharide binding pocket visualization; pH6 antigen–lactose binding pocket visualization; Galectin-3C–lactose binding pocket visualization; MM/GBSA monosaccharide energetics; MM/GBSA disaccharide energetics; WT-MetaD *versus* MM/GBSA total energy comparisons; galectin-3C MM/GBSA residue decomposition energetics; cholera toxin MM/GBSA residue decomposition energetics; pH6 antigen MM/GBSA residue decomposition energetics; progenitor toxin MM/GBSA residue decomposition energetics; galectin-10 MM/GBSA residue decomposition energetics; QM cluster model preparation; QM cluster model calculation; tabulated energetics for intermediates and transition states; translocation QM cluster model contents; NEB potential energy surface with all residues; NEB potential energy surface with only tryptophan; energy profile for translocation with and without protein residues (PDF).

Protein master list with structure information and sequence clustering information; input files for protein simulations; structures and energetics of the QM cluster models of translocation (Zenodo).
